# Response of stratospheric water vapour to warming constrained by satellite observations

**DOI:** 10.1038/s41561-023-01183-6

**Published:** 2023-06-26

**Authors:** Peer Nowack, Paulo Ceppi, Sean M. Davis, Gabriel Chiodo, Will Ball, Mohamadou A. Diallo, Birgit Hassler, Yue Jia, James Keeble, Manoj Joshi

**Affiliations:** 1 Climatic Research Unit, School of Environmental Sciences, University of East Anglia, Norwich, UK; 2 Grantham Institute and Department of Physics, Imperial College London, London, UK; 3 Data Science Institute, Imperial College London, London, UK; 4 Institute of Theoretical Informatics, Karlsruhe Institute of Technology, Karlsruhe, Germany; 5 NOAA Chemical Sciences Laboratory, Boulder, CO USA; 6 Institute for Atmospheric and Climate Science, ETH Zurich, Zurich, Switzerland; 7 Department of Geoscience and Remote Sensing, Delft University of Technology, Delft, The Netherlands; 8 Physikalisch-Meteorologisches Observatorium Davos World Radiation Centre, Davos, Switzerland; 9 Institute of Energy and Climate Research, Stratosphere (IEK-7), Forschungszentrum Jülich, Jülich, Germany; 10 Deutsches Zentrum für Luft- und Raumfahrt (DLR), Institut für Physik der Atmosphäre, Oberpfaffenhofen, Germany; 11 Cooperative Institute for Research in Environmental Sciences (CIRES), University of Colorado Boulder, Boulder, CO USA; 12 Yusuf Hamied Department of Chemistry, University of Cambridge, Cambridge, UK; 13 National Centre for Atmospheric Science (NCAS), University of Cambridge, Cambridge, UK

**Keywords:** Climate and Earth system modelling, Projection and prediction, Atmospheric science

## Abstract

Future increases in stratospheric water vapour risk amplifying climate change and slowing down the recovery of the ozone layer. However, state-of-the-art climate models strongly disagree on the magnitude of these increases under global warming. Uncertainty primarily arises from the complex processes leading to dehydration of air during its tropical ascent into the stratosphere. Here we derive an observational constraint on this longstanding uncertainty. We use a statistical-learning approach to infer historical co-variations between the atmospheric temperature structure and tropical lower stratospheric water vapour concentrations. For climate models, we demonstrate that these historically constrained relationships are highly predictive of the water vapour response to increased atmospheric carbon dioxide. We obtain an observationally constrained range for stratospheric water vapour changes per degree of global warming of 0.31 ± 0.39 ppmv K^−1^. Across 61 climate models, we find that a large fraction of future model projections are inconsistent with observational evidence. In particular, frequently projected strong increases (>1 ppmv K^−1^) are highly unlikely. Our constraint represents a 50% decrease in the 95th percentile of the climate model uncertainty distribution, which has implications for surface warming, ozone recovery and the tropospheric circulation response under climate change.

## Abstract (web summary)

Uncertainty in stratospheric water vapour projections is reduced using an observational constraint based on historical co-variations between atmospheric temperature and water vapour.

## Main

The stratosphere is extremely dry. This was first realized by Alan Brewer in his pioneering analysis of balloon measurements in the 1940s, where he reported that the atmospheric *water content is found to fall very rapidly just above the tropopause*^1^. It is now well established that average stratospheric specific humidity is around 3–5 parts per million volume (ppmv) globally, with substantial daily to decadal variations driven by volcanic eruptions^2,3^, convective overshooting^4^, monsoonal circulations^5^ and climate modes such as the El Niño-Southern Oscillation (ENSO)^6,7^ and the Quasi-Biennial Oscillation (QBO)^8,9^. Brewer also already suggested that the dryness of the stratosphere can be explained by a large-scale stratospheric overturning circulation nowadays referred to as the Brewer–Dobson circulation (BDC)^10^, where air is freeze dried to very low concentrations as it enters the stratosphere through the cold tropical upper troposphere and lower stratosphere (UTLS).

The freeze-drying process is now better understood than ever, for example, using air parcel trajectory models of the tropical UTLS^5,11–16^. However, despite this qualitative understanding, there is still substantial model uncertainty in projections of future changes in stratospheric water vapour (SWV)^17–21^. The uncertainty is a pressing concern, not only because SWV is a greenhouse gas affecting surface temperature and the atmospheric circulation^22–26^, but also because of its key role in shaping atmospheric chemistry and stratospheric ozone recovery^27–29^. Changes in the thickness of the ozone layer, in turn, affect the tropospheric photochemical environment, air quality, human health and ecology^30,31^.

Historically, tropical lower SWV observations show—if anything—a slight decrease over the last three decades (Supplementary Fig. 1; refs. ^15,32^), at least until the recent Hunga–Tonga eruption^33^. In contrast, the majority of climate models show long-term increases in historical simulations (Supplementary Figs. 1 and 2; refs. ^17,21^). Given substantial model biases in background concentrations and seasonal cycle representations of SWV and UTLS temperatures^17,21^, one might therefore ask if these models can reliably project SWV for future scenarios. In addition, it is unclear if models that better match observations^17,18^ can be trusted more in their projections because past biases do not always translate into future projections^21,34^.

**Fig. 1:**
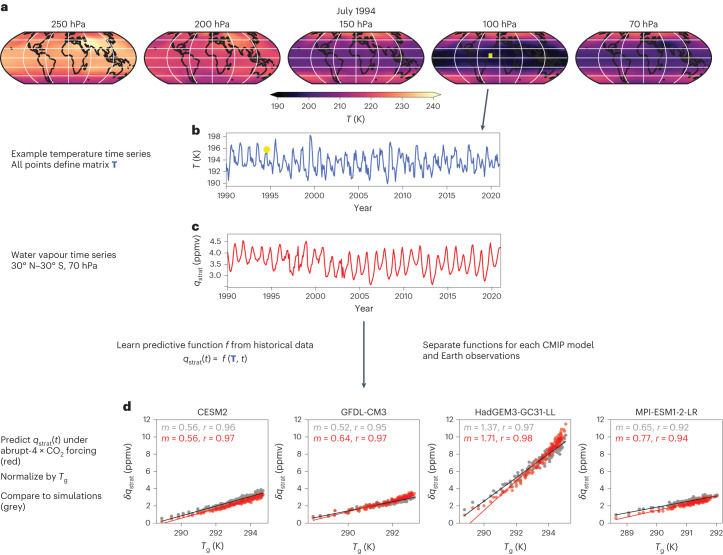
Sketch of the statistical-learning framework. **a**, Example ERA5 reanalysis temperature (*T*) data from the European Centre for Medium-Range Weather Forecasts (ECMWF) for July 1994 at all five pressure levels used to predict tropical lower SWV (*q*_strat_). **b**, Example of a temperature time series for the grid location highlighted in yellow on the 100 hPa map in **a**; the yellow dot indicates the July 1994 value. All 5° × 5° grid points within 60° N–60° S serve as predictors for *q*_strat_(*t*), defining a temperature matrix **T**(*t*). **c**, The observational *q*_strat_(*t*) time series. Using ridge regression, we learn predictive relationships between **T** covering all five pressure levels and *q*_strat_, considering simultaneous and lagged temperature data (${\tau }_{\max }$ = 2 months). This process is applied to 150 combinations of temperature reanalyses and versions of *q*_strat_ observations from 1990 to 2020 (Methods). The result is an ensemble of predictive functions that are consistent with the observational record. We then learn equivalent functions from data produced by 27 climate models from the CMIP5/6 archives, that is, using data of the same spatial and temporal coverage for **T** and *q*_strat_. **d**, For these CMIP models, the predictive skill of the functions can be evaluated under strong climate change scenarios. As illustrated here for four CMIP models, this is achieved by comparing the annually averaged predictions (red) with actual abrupt-4 × CO_2_ simulation results (grey). From these comparisons, a framework-related uncertainty is estimated characterizing imperfections in the statistical-learning predictions under 4 × CO_2_, which needs to be smaller than the model uncertainty to be constrained. To account for different levels of global warming simulated by each model, the *q*_strat_ response (in ppmv; here shown relative to average 1990–2020 levels) is normalized by the change in global mean surface temperature (*T*_g_). Colour-coded inset values for *m* represent the linear regression slopes (ppmv K^−1^) and *r* the correlation coefficients.

Here we introduce a statistical-learning framework to derive an observational constraint on these uncertain model projections. We estimate high-dimensional regression functions to predict tropical lower SWV from the UTLS temperature structure (Fig. 1), given the aforementioned link between UTLS temperatures and tropical dehydration. UTLS temperatures integrate the effects of a large number of processes affecting air dehydration, either directly or indirectly^8,15,18,35^. Our primary interest therefore is to quantify known relationships between the UTLS temperature structure and tropical lower SWV^9,36^ but in a novel way that allows these relationships to hold under strong climate change scenarios. This, in turn, will open up new pathways for estimating observational constraints on future projections. Our analysis will not consider SWV production from changes in stratospheric methane concentrations, which gains importance in the middle to upper stratosphere^37,38^. To minimize such influences on our results, we focus on the tropical lower stratosphere at 70 hPa, that is, water vapour just above the tropical cold-trap region where air dehydration takes place^39^.

## Learning predictive relationships from historical data

We aim to learn predictive functions *f*, ultimately characterized by their coefficients **Θ**

1 $$\log \left({q}_{{{{\rm{strat}}}}}(t)\right)=f({{{\boldsymbol{\Theta }}}},{{{\bf{{T}}}}};t,{\tau }_{\max })=\mathop{\sum }\limits_{{{{{i}}}}}^{{{{\rm{lat}}}}}\mathop{\sum }\limits_{{{{{j}}}}}^{{{{\rm{lon}}}}}\mathop{\sum }\limits_{{{{{k}}}}}^{{{{{p}}}}}\mathop{\sum }\limits_{\tau }^{{\tau }_{\max }}{{{\Theta }}}_{{{{{ijk}}}},\tau }{{{\rm{d}}}}{T}_{{{{{ijk}}}}}\left(t-\tau \right)$$

which predict 30° N–30° S average, monthly and zonal mean SWV (specific humidity, in ppmv) at 70 hPa, mimicking frequently used indices characterizing water vapour entry rates through the tropical cold-trap region^17,18,35^. Hereafter, we will refer to this quantity as *q*_strat_. d*T*_ijk_ is the standard-scaled monthly mean temperature (that is, zero meaned and scaled by its own *σ* over the training period) at 5° × 5° latitude–longitude grid points indexed by (*i*,*j*) within one of *p* = 5 atmospheric levels (250, 200, 150, 100, 70 hPa) indexed by *k*, covering a latitudinal range of 60° N–60° S (Fig. 1a).

**Extended Data Fig. 1:**
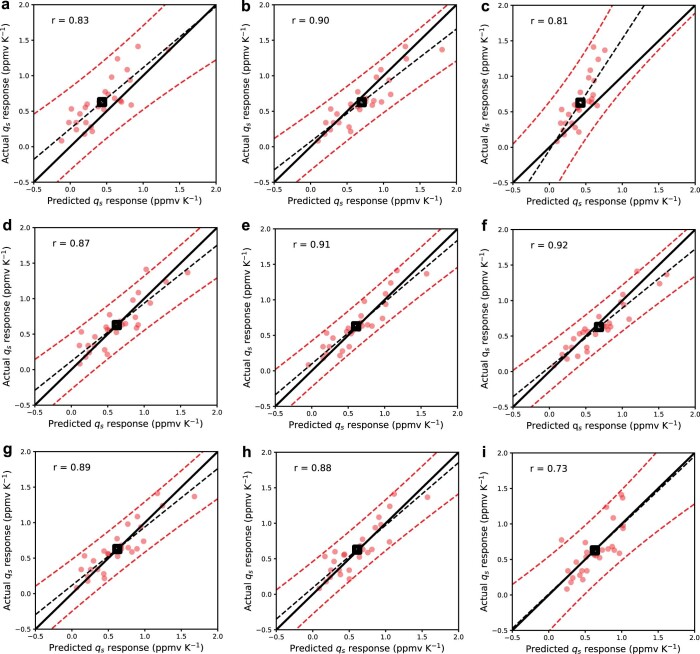
Framework performance depending on regression settings. As Fig. 2a, that is red circles show abrupt-4xCO_2_ simulation results (’actual’) regressed against predicted changes in *q*_strat_ (here abbreviated as *q*_*s*_), both normalized by *T*_*g*_, for 27 CMIP models. The multi-model-mean is indicated as a black square; the one-to-one line in solid black. Dashed lines show the least squares regression fit (black) and the 5 to 95% prediction intervals (red). The one-at-a-time differences are that in **a** no lagged temperature data was considered as predictors; in **b** one additional time lag (${\tau }_{\max }=3$) was considered; in **c** we did not take the natural logarithm of *q*_strat_; in **d** temperature predictors at all latitudes were considered; in **e** temperature predictors only within 30^∘^N - 30^∘^S were considered; and in **f** 444 samples (months covering all years from 1984 to 2020) were used for training the CMIP functions, instead of the 315 months used in the main paper. In **g**, temperature data at seven pressure levels (300, 250, 200, 150, 100, 70, 50 hPa) were considered as predictors, whereas in **h** only three levels (200, 150, 100 hPa) and in **i** temperature only at 100 hPa was considered.

*f* predicts *q*_strat_ at time *t* with high skill and we cross-validated its performance for various regression specifications (Extended Data Fig. 1). These tests included the choice of pressure levels, latitude range and number of time lags for **T**. Unsurprisingly, we found that predictive performance improves if we also consider temperature data from the two preceding months (that is, ${\tau }_{\max }=2$ months), being reflective of the slow vertical ascent of air through the tropical tropopause layer^12,35^. We additionally apply logarithmic transformations to the *q*_strat_ data as to approximately account for nonlinearity in the *T*–*q*_strat_ relationships. A central concern when learning such high-dimensional regression functions from a relatively small number of (observed) monthly samples is to avoid overfitting. To manage this issue, we here use ridge regression^40^, similar to an approach recently applied successfully to constrain the global cloud feedback on climate change^41^.

We then learn different functions *f* from sets of **T** and *q*_strat_ time series from both observations and climate models (Fig. 1b,c). As proxies for observations, we use the Stratospheric Water and OzOne Satellite Homogenized (SWOOSH)^42^ *q*_strat_ dataset and three reanalysis products for temperature and remove months when *q*_strat_ observations are missing or unreliable (for example, related to the Mount Pinatubo eruption; Methods). The use of multiple reanalysis products and of SWOOSH uncertainty estimates allows us to incorporate the effects of measurement uncertainty in our constraints. For climate model data, we use simulations covering the same historical period or slightly shifted (depending on data availability) from the Coupled Model Intercomparison Project phases 5 and 6 (CMIP5/CMIP6; Methods). We treat each CMIP dataset in the same way as SWOOSH by masking equivalent months. Many models do not achieve realistic amplitudes of *q*_strat_ variability^17,21^, leading to insufficiently clear signals for ridge regression to learn from. We therefore sub-select 27 models that at least approximate the SWOOSH variance (Methods). This selection is still meant to sample the model uncertainty in the *T*_*ijk*_ and *q*_strat_ responses across CMIP models so that our observational constraint will be based on better estimates for the learned parameters **Θ**.

We obtained high predictive skill for each *f* on historical time slices not used during training and cross-validation (typically *r*^2^ scores > 0.8). However, this is unsurprising given the central role temperature plays in setting present-day *q*_strat_. More challenging is the aim to use the functions trained on historical data to predict *q*_strat_ responses under increased greenhouse gas forcing, that is, that the past relationships also hold in significantly warmer climates. Such climate-invariant functions open up new pathways to observationally constrain the *q*_strat_ response to climate change. We note that previous studies developed climate index-based multiple linear regression (MLR) methods to analyse SWV variability and trends in observations and models^9,15,18,35,43,44^. On the basis of small sets of indices, these valuable tools can explain large fractions of variance and have been used to infer drivers of SWV changes under climate forcing^18^. However, these MLR methods do not achieve the predictive performance of ridge regression under extrapolation (Supplementary Fig. 3), underlining the value of statistical learning for deriving our observational constraint. We further see advantages in exploiting only well-observed UTLS temperatures as predictors, whereas index-based methods typically require BDC metrics that are not a widely available CMIP output, allowing for evaluation of a greater number of models.

## The observational constraint

We evaluate the extrapolation idea in a perfect-model setting: the 27 functions trained on historical CMIP data are used to predict *q*_strat_ under abrupt-4 × CO_2_ forcing, using the ${{\mathbf{T}}}_{4\,\times\,{{{{\rm{CO}}}}}_{2}}$ from the corresponding CMIP simulations as predictors. In Fig. 1d, we show four examples of such comparisons between statistical-learning predictions (red) and actual 4 × CO_2_ simulation results (grey; Supplementary Figs. 4 and 5 provide all 27 model results). To enable comparisons across models with very different climate sensitivities, we normalize annually averaged *q*_strat_ trends by the model-specific changes in global mean surface temperature (*T*_g_), as is common in climate-feedback analyses, for example, ref. ^41^, resulting in *q*_strat_ trends per degree of global warming (ppmv K^−1^). This choice is justified by the close coupling between UTLS temperatures and surface warming (Supplementary Figs. 4 and 5). However, as an alternative viewpoint, we provide equivalent results for a normalization by zonal mean temperatures close to the cold-trap region (20° N–20° S, 100 hPa) in Supplementary Figs. 6–8.

**Fig. 2:**
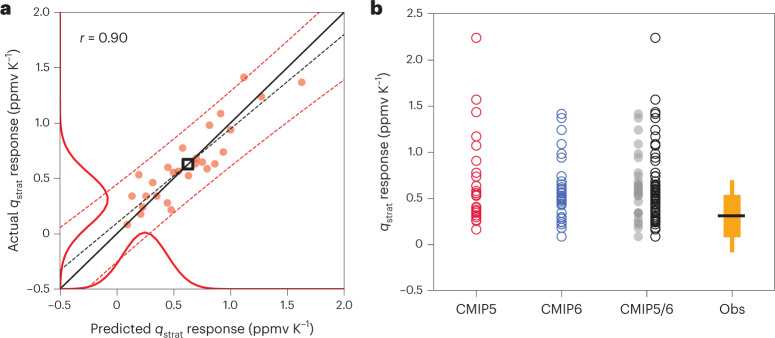
Framework performance and the observational constraint. **a**, Red circles show abrupt-4 × CO_2_ simulation results (‘actual’) regressed against predicted changes in *q*_strat_, both normalized by *T*_g_, for 27 CMIP models. The multi-model mean is indicated as a black square; the one-to-one line in solid black. Dashed lines show the least squares regression fit (black) and the 5% to 95% prediction intervals (red). The probability distributions (red curves) on the axes represent the observational estimates. The distribution on top of the *x* axis indicates the spread in predictions based on combining functions learned from observations with the CMIP temperature responses. The final probability distribution, defining the observational constraint, is attached to the *y* axis and additionally accounts for the framework prediction uncertainty. **b**, The observational constraint (*n* = 4,050) relative to CMIP model uncertainty. Circles show *T*_g_-normalized changes in *q*_strat_ for 27 CMIP5 models (red), 34 CMIP6 models (blue) and their combination (black). The grey circles indicate the selected 27 models fulfilling the minimum variance criterion compared with SWOOSH used for the framework validation in **a**. The observational constraint (orange; Obs) is illustrated on the right with the horizontal black line indicating the 50th percentile (0.31 ppmv K^−1^). The thin and thick bars denote 90% (−0.09 to 0.69 ppmv K^−1^) and 66% (0.08 to 0.54 ppmv K^−1^) confidence intervals, respectively. The CMIP mean (median) values are 0.67 (0.53) ppmv K^−1^ for CMIP5, 0.55 (0.49) ppmv K^−1^ for CMIP6, 0.60 (0.52) ppmv K^−1^ for the combined set of CMIP5/6 and 0.63 (0.59) ppmv K^−1^ for the 27 selected models.

Comparing the predictions to the actual CMIP results, we find excellent agreement across the multi-model ensemble (*r* = 0.90; Fig. 2a), indicating that the historical *T*–*q*_strat_ relationships also hold well under strong greenhouse gas forcing and opening up a path to an observational constraint in three steps: (1) Given three reanalysis datasets and *n* = 50 iterations of SWOOSH *q*_strat_ time series with varying added noise patterns (Methods; to estimate sampling and measurement uncertainty), we arrive at 3 × 50 = 150 statistical-learning functions ***f***_obs_ consistent with the observational record, each with its own set of coefficients **Θ**_obs_. (2) Using these ***f***_obs_, we combine the uncertainty contributions introduced due to spread in the **Θ**_obs_ and in the CMIP temperature responses ${\mathbf{T}}_{4\,\times\,\mathrm{CO}_2}$ (Methods), leading to a probability distribution for the observational prediction (shown along the *x* axis of Fig. 2a; solid red curve). (3) Finally, this distribution is convolved (Methods) with the framework-intrinsic prediction error evident from the scatter around the one-to-one line in Fig. 2a. The result is the probability distribution characterizing the observational constraint (shown along the *y* axis).

Figure 2a demonstrates the large spread across the CMIP *q*_strat_ responses (0.08 to 1.41 ppmv K^−1^), as compared to 0.31 ± 0.39 ppmv K^−1^ (90% confidence interval) for our observational constraint. The large model uncertainty is illustrated even more clearly in Fig. 2b for 27 CMIP5 models (red), 34 CMIP6 models (blue), the combined CMIP5/6 ensemble (black) and for the selected 27 CMIP5/6 models (grey). Ten CMIP models simulate trends of approximately 1.0 ppmv K^−1^ or larger, that is, well outside the observationally plausible range (orange). While the median across all 61 CMIP models (0.52 ppmv K^−1^) is still within typical uncertainty bounds, a substantial number of models are highly likely to overestimate the *q*_strat_ feedback under global warming. Considering typical confidence intervals for our constraint with 0.08 to 0.54 ppmv K^−1^ (17% to 83%), −0.09 to 0.69 ppmv K^−1^ (5% to 95%) and −0.17 to 0.77 ppmv K^−1^ (2.5% to 97.5%), we find that about one-fifth (13) of the models exceed even the 97.5th percentile of the constraint and almost half of the models (27) exceed the 83rd percentile (upper end of the thick orange bar in Fig. 2b).

Notably, the observational constraint includes small negative *q*_strat_ responses not seen in the models. It is probably unsurprising that negative feedbacks cannot be entirely ruled out given historically (rather) negative trends under global warming, limited sample size and possible external interferences not removed by data pre-processing (for example, remaining effects of volcanic eruptions). A negative SWV trend could also be driven by a strong BDC response, which would act to cool the tropical UTLS under CO_2_ forcing^15,18,35^. The fact that we find a robustly positive 50th percentile for the constrained response underlines our hypothesis that the framework does not merely reproduce historical trends but can indeed learn approximately climate-invariant *T*–*q*_strat_ relationships *from* internal variability (for example, related to QBO or ENSO) instead of being negatively impacted *by* it.

## Emulation of the historical record and inference

**Fig. 3:**
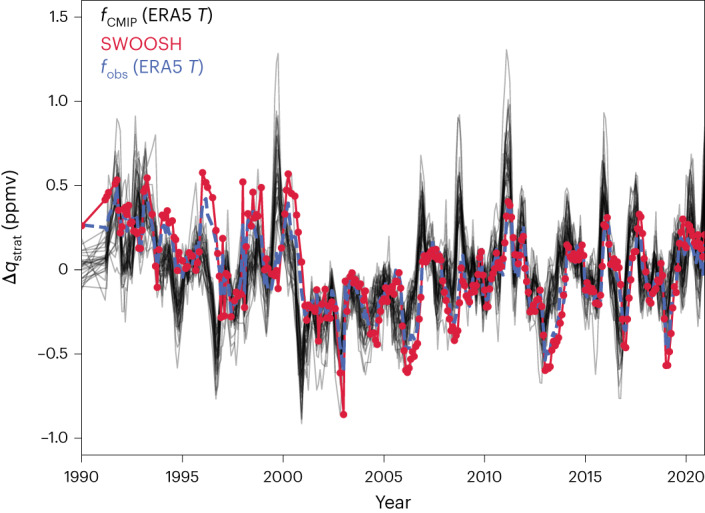
CMIP-based predictions of past variability in tropical lower stratospheric water vapour. Black: monthly mean predictions of deseasonalized Δ*q*_strat_ anomalies using the 27 CMIP-based functions provided with ERA5 reanalysis temperature data. We also show SWOOSH observational data for the same period (red). The blue line indicates average predictions conducted with the cross-validated functions learned from SWOOSH and ERA5, if ERA5 temperatures are used again as input. These predictions are highly correlated with SWOOSH (*r*^2^ score = 0.90; Pearson’s *r* = 0.96). The CMIP-based predictions also correlate well with SWOOSH but typically overestimate the amplitude of the undulations in line with their too-large sensitivities under climate change. Predictions with other reanalysis temperatures provide similar results (Extended Data Fig. 2).

**Extended Data Fig. 2:**
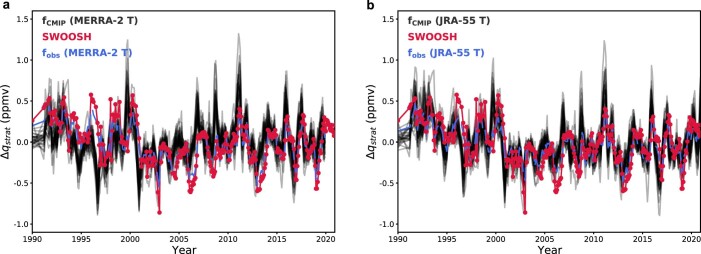
CMIP-based predictions of past variability in tropical lower stratospheric water vapour using two other reanalysis temperature datasets. Black: monthly mean predictions of past Δ*q*_strat_ anomalies (relative to the respective seasonal cycles), using the CMIP-based functions provided with **a** MERRA-2 and **b** JRA-55 temperature data. We also show SWOOSH observational data for the same period (red), with the dots indicating the timing of the 315 months used in our calculations. The same months were selected from MERRA-2/JRA-55 for the CMIP-based *q*_strat_ predictions. The blue dashed line indicates the averaged predictions using the cross-validated ridge functions learned from the 50 combinations of SWOOSH and MERRA-2/JRA-55 data, if MERRA-2/JRA-55 is used again as the consistent input. The comparison with the SWOOSH time series (red) itself underlines that these ridge regressions represent a large fraction of the SWV variance, as evident from high r^2^ scores of 0.89 for **a** and 0.79 for **b** (see for example ref. ^58^ for a detailed explanation of this time series performance metric) and Pearson correlation coefficients of 0.96 and 0.90, respectively.

We now ask if CMIP models are, in principle, able to reproduce observed *q*_strat_ variability. We use the 27 functions trained on CMIP data to emulate the historical record of *q*_strat_ anomalies, given temperature data from the reanalyses as predictors (Fig. 3 and Extended Data Fig. 2). These CMIP-based predictions of the historical record (black) are compared to SWOOSH (red). We find that the observed variations, including the sudden drop in *q*_strat_ in the year 2000 (refs. ^25,45^), are captured relatively well. The implication is that if provided with realistic UTLS temperature fields, most CMIP models would display the correct *q*_strat_ tendencies. However, we also highlight that the year-to-year variability of the statistical-learning predictions typically takes on amplitudes substantially larger than those observed, in agreement with our key result of overly sensitive *T*–*q*_strat_ relationships already seen in their abrupt-4xCO_2_ responses.

**Fig. 4:**
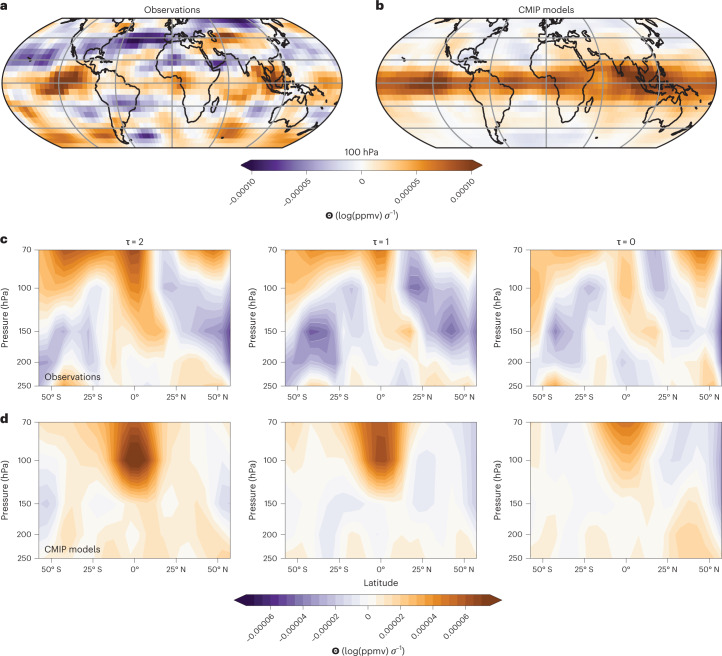
Interpretation of the statistical-learning results. **a**, Ridge coefficients **Θ** at 100 hPa and for lag *τ* = 2 months averaged over all 150 observational functions. **b**, The same for the multi-model mean of the 27 CMIP models. The predictor temperature data were standard scaled for each grid point (units log(ppmv) *σ*^−1^). The **Θ** magnitudes are therefore also directly comparable, that is, larger positive coefficients imply a greater humidifying effect for a typical local temperature increase. **c**,**d**, Zonal mean **Θ** for observations (**c**) and for the CMIP multi-model mean (**d**). The latitude–height cross sections illustrate an upward propagation of the sources of predictive information over time, reflective of the slow ascent of air through the tropical UTLS.

To detect the origin of these overestimated *T*–*q*_strat_ sensitivities, we highlight the use of the statistical-learning functions for understanding model–observation discrepancies, for example, by visualizing the parameters **Θ** (Fig. 4 and Supplementary Figs. 9–11). This is possible because in ridge regression the absolute magnitude of each coefficient is proportional to its estimated prediction importance, that is, larger size coefficients imply greater importance. While interpreting these coefficient maps is non-trivial, we point out a few emerging patterns. For **Θ**_obs_, independent of the reanalysis dataset used, we, for example, find at 100 hPa (Fig. 4a) and below features suggestive of influences by tropical circulation anomalies, possibly ENSO^7,9,18^, in particular around the Maritime Continent and above the Eastern Pacific. The largest coefficients occur in a narrow band across the inner tropics (10° N–10° S) from 100 hPa upwards in agreement with the well-understood slow vertical ascent of tropical air masses through this cold-trap region (Fig. 4a,b). Zonal mean latitude–height cross sections of **Θ** show a clear upward progression of predictive information over time (Fig. 4c,d), supporting the view that the functions correctly identify the main characteristics of the underlying coupling between the large-scale circulation and tropical UTLS dehydration. Crucially, the CMIP multi-model mean **Θ** (Fig. 4b,d) strongly overestimates the inner tropical relationships between *T* and *q*_strat_, underlining the over-sensitivity of CMIP models on average. For CMIP models, the peak positive inner tropical **Θ** (probably representing the tape-recorder signal^36^), additionally maximizes at 100 hPa without further growth with altitude, contrary to the **Θ**_obs_. We speculate that this discrepancy could be caused by the low vertical resolution of many CMIP models around the tropical tropopause. The **Θ** maps also uncover a few other intriguing discrepancies, including a pattern of large negative **Θ**_obs_ at 100 hPa across the North Atlantic, which is part of a general strong positive to negative, tropics to extratropics gradient in **Θ**_obs_ not reproduced in the CMIP mean. However, similar, or even clearer, patterns do occur in many individual models (Supplementary Figs. 12–15). A reason might be the modulating role of the subtropical jet streams and their induced mixing barriers on tropics–extratropics SWV exchange whose strength will also depend on UTLS temperature gradients. To test such hypotheses, and to distinguish significant patterns in the coefficients from noise, we below recommend future modelling experiments to design systematically perturbed datasets to train ridge regressions on.

## Constraint on the radiative feedback and implications

In conclusion, we have derived an observational constraint for changes in tropical lower SWV per degree global warming of 0.31 ± 0.39 ppmv K^−1^ (90% confidence interval). This constraint on current modelling uncertainty has important implications for the stratospheric feedback onto climate change^19,20,26^ and for the recovery of the stratospheric ozone layer^27–29^. Indeed, our framework opens up new routes to the process-oriented evaluation of, and observational constraints on, state-of-the-art climate model projections. As such, we recommend its use as a complement to easily interpretable, but only analytically applicable, climate index-based regressions^9,15,18,35,43^.

Our observational constraint is possible only through a highly effective statistical-learning approach to estimate climate-invariant relationships between UTLS temperatures and SWV from the still very limited record of SWV observations. In a perfect-model setting, we have confirmed that these relationships also seem to hold under large CO_2_ forcing and are robust to the presence (or absence) of, for example, historical changes in aerosol and methane-related interferences. Our results reveal a widespread over-sensitivity in CMIP models of tropical lower SWV to changes in UTLS temperatures. In particular, our constraint implies that frequently modelled large increases per degree global warming > 1 ppmv K^−1^ are highly unlikely. Strikingly, around a quarter of CMIP models exceed even the upper 95th percentile of our constraint. Given the 90% range of model responses (0.18–1.41 ppmv K^−1^), our constraint represents a 50% decrease in the 95th percentile of the climate model uncertainty distribution and a narrowing of 37% of the overall 90% range (which includes small negative responses not found in CMIP).

**Extended Data Fig. 3:**
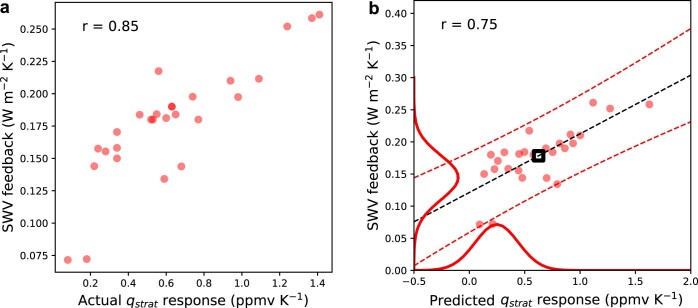
Constraint on the stratospheric water vapour feedback parameter. **a** Correlations of the radiative feedback parameters for the 27 models also used in Fig. 2a in the main text against the *q*_strat_ metric, yielding a high correlation. **b** As Fig. 2a, but again with the radiative feedback parameters instead, leading to an observational constraint. The final distribution is shown along the *y*-axis.

Whereas the 27 models exhibit a wide range of total radiative SWV feedbacks of 0.091–0.256 W m^−2^ K^−1^ (90% confidence interval, with a median of 0.18 W m^−2^ K^−1^; Methods and Supplementary Table 1), we can apply our constraint on tropical lower SWV to also constrain this uncertainty by 30% to 0.086–0.201 W m^−2^ K^−1^ (median = 0.14 W m^−2^ K^−1^; Extended Data Fig. 3). This estimate represents an uncertainty reduction of 0.05 W m^−2^ K^−1^, which is comparable to the effects of changes in biogenic volatile organic compounds or ozone (table 6.8 in ref. ^46^) and thus of relevance to policymakers. Our work further opens up new pathways for constraining the effects of changes in SWV on catalytic ozone-depletion cycles^28,29^, Arctic amplification, the North Atlantic Oscillation, the stratospheric circulation and the tropospheric jet streams^24,26^.

Finally, we highlight the urgent need to identify and address model-dependent root causes of the over-sensitivity, such as UTLS temperature biases^7,17,21^, atmospheric chemical feedbacks^19^, QBO influences on UTLS temperature variability^8^ or unrealistic diffusivity of water vapour across the tropical tropopause^47^. Such efforts might benefit from analyses of how a variety of stratospheric (de-)hydration mechanisms^4,39^ affect results within our novel observational constraint framework, for example, by learning from data produced in perturbed-physics ensembles or following targeted climate model tuning^48,49^. In particular, processes that might not (yet) be included in climate models pose potential blind spots to our perfect-model validation approach (Fig. 2a). We also highlight that our framework could be extended to address other key uncertainty factors in stratospheric climatology and atmospheric chemistry. A non-exhaustive list includes extratropical SWV trends, especially those in the radiatively important lowermost stratosphere^2,20,50^, and trends in lower stratospheric ozone^19^.

## Methods

### Water vapour observations and their uncertainty estimates

For SWV observations, we use the global Stratospheric Water and OzOne Satellite Homogenized (SWOOSH)^42^ dataset, which includes vertically resolved water vapour data from a subset of the limb-profiling satellite instruments operating since the 1980s. SWOOSH is designed to accurately reproduce monthly average variability present in the underlying data. We select the variable *combinedanomfillh2oq* at 68 hPa, which is an anomaly-filled zonal mean specific humidity field in parts per million volume (ppmv) at 10° latitude resolution. We spatially weight (cosine-weighting for latitudes) and average (latitude–longitude) the field for points within 30° N to 30° S to obtain a representation of tropical lower SWV.

For our analysis, we consider SWOOSH v2.7 data covering the period from January 1984 to including December 2020. The version incorporates recent improvements in Earth Observing System Aura Microwave Limb Sounder (MLS) data^51^. However, before the availability of Aura MLS satellite data (September 2004–present), the SWOOSH dataset has a high number of missing data in the tropics. For the anomaly-filled version of SWOOSH, missing data were filled using a procedure that made 2D latitude–time linear interpolations for each month on deseasonalized anomalies using information from adjacent grid cells for which data existed; the seasonal cycle was added back on after filling in the latitude–time plane at each pressure level^42^. However, the interpolation was not evaluated for potential biases introduced by the procedure so that the use of the filled product for our statistical-learning approach introduces an additional uncertainty factor.

Here we make a first-order estimate of additional biases for the pre-MLS period by using the MLS period where sampling is high and effectively unbiased by latitude and time and masking these data as for the pre-MLS period. For each month for which at least one sample exists in the 30° S and 30° N latitude band in the pre-MLS period, we identify the month of that year in all Aura MLS data, mask the data as for the month of interest and estimate the bias introduced by integrating, weighting by latitude over 30° S to 30° N where data exist, and by comparing with the ‘true’ unbiased MLS/SWOOSH value. From this, we estimate a mean and standard deviation of the bias assuming Gaussianity.

For our final uncertainty calculations, we dropped all months from the SWOOSH dataset for which there was not at least one sample measured within 30° S to 30° N, which reduces the total number of samples (months) considered from 444 to 315. In effect, this also removes all SWOOSH data before January 1990. For the remaining 315 months, we estimate the uncertainty introduced by the biases of the anomaly-filling method by sampling (in addition to the uncertainty provided by SWOOSH and assuming a normal distribution) from the standard deviation in the MLS bias estimates outlined above and adding this to a random normal sample from the standard error of the SWOOSH data itself, that is, *σ*/$\sqrt{N}$, latitude-weighted by the sum of the squared errors. Here *N* is the overall measured number of samples per monthly data point considered^42^. Adding these randomly drawn estimates for each month to the original filled SWOOSH time series yields sets of ‘sampled time series’. Here we use *n* = 50 such randomly drawn time series to estimate the effects of the sampling biases on our overall uncertainty estimates. We find that the effect of sampling biases are small to negligible for the overall uncertainty estimation but we still include these error estimates in our uncertainty analysis for completeness.

### Temperature data

To approximate observations for UTLS temperatures, we use three different reanalysis datasets for temperature at 250, 200, 150, 100 and 70 hPa over the same time period: ERA5 (ref. ^52^), MERRA-2 (ref. ^53^) and JRA-55 (ref. ^54^). For MERRA-2, we do not include the year 2020 as the corresponding data could not be found in the archive used at the time of writing (Data Availability). For learning the observational constraint functions, we combine each of the three reanalyses once with each of the *n* = 50 SWOOSH randomly drawn time series, resulting in 150 functions overall. From these 150 functions, we derive a first observational uncertainty estimate on predictions under 4 × CO_2_ forcing by providing each function once with the modelled (and standard-scaled) monthly mean temperature profiles found under 4 × CO_2_ for the 27 selected CMIP models (steps also described in the main text).

### CMIP data

We consider climate model data from both the CMIP5 (ref. ^55^) and CMIP6 (ref. ^56^) archives. In total, this amounted to 61 models for which we found data for all required scenarios:

- 27 CMIP5 models: ACCESS1-0, ACCESS1-3, BCC-CSM1-1, BCC-CSM1-1-m, BNU-ESM, CanESM2, CCSM4, CNRM-CM5, CSIRO-Mk3-6-0, EC-EARTH, FGOALS-g2, GFDL-CM3, GFDL-ESM2G, GFDL-ESM2M, GISS-E2-H, GISS-E2-R, HadGEM2-ES, INM-CM4, IPSL-CM5A-MR, IPSL-CM5B-LR, MIROC5, MIROC-ESM, MPI-ESM-LR, MPI-ESM-MR, MPI-ESM-P, MRI-CGCM3, NorESM1-M.
- 34 CMIP6 models: ACCESS-CM2, ACCESS-ESM1-5, AWI-CM-1-1-MR, BCC-CSM2-MR, BCC-ESM1, CAMS-CSM1-0, CanESM5, CESM2, CESM2-WACCM, CNRM-CM6-1, CNRM-ESM2-1, E3SM-1-0, EC-Earth3-Veg, FGOALS-f3-L, FGOALS-g3, GFDL-CM4, GFDL-ESM4, GISS-E2-1-G, GISS-E2-1-H, HadGEM3-GC31-LL, HadGEM3-GC31-MM, INM-CM4-8, INM-CM5-0, IPSL-CM6A-LR, MIROC6, MIROC-ES2L, MPI-ESM1-2-HR, MPI-ESM1-2-LR, MRI-ESM2-0, NESM3, NorESM2-LM, NorESM2-MM, SAM0-UNICON, UKESM1-0-LL.

An overview of all CMIP models, including individual model references and *T*_g_-normalized *q*_strat_ feedback values, is provided in Supplementary Table 2. Equivalent results for normalization by 20° N –20° S temperature at 100 hPa are tabulated in Supplementary Table 3. For each model, we used variable output for 30° S–30° N average zonal mean specific humidity (hus) at 70 hPa and air temperature (ta) at 250, 200, 150, 100 and 70 hPa. To train the ridge regressions, we combined atmosphere–ocean-coupled historical simulations from 1 January 1984 onwards with Representative Concentration Pathway 4.5 (RCP4.5)/Shared Socioeconomic Pathway 3–7.0 (SSP3–7.0) scenarios. The future RCP scenarios were selected as to maximize the number of models for which we could match the observed period within either CMIP archive given that scenario differences across the period 2005 (end of historical simulations for CMIP5) to 2020 (end of observed period used here) are negligible for our calculations. The same two variables plus surface air temperature (tas) were extracted for the same set of models for the abrupt-4 × CO_2_ simulations. In all cases, we use only the first available ensemble member for each model.

It is well known that tropical UTLS water vapour variability is not represented well in many atmospheric models, both in terms of the timing and amplitude of the seasonal cycle and/or variations relative to it^17,21^ (Supplementary Figs. 1 and 2). A concern of particular importance for the statistical-learning process employed here are cases where variability is substantially underestimated, because this will reduce the ability of ridge regression to learn meaningful *T*–*q*_strat_ relationships, especially if the goal is to extrapolate potentially very large abrupt-4 × CO_2_ responses^41,57,58^. We therefore include only CMIP models that represent at least 95% of the observed variance found for SWOOSH across the 315 potential training samples in our calculations (Fig. 2a) in the main text. These 27 models are:

- Six CMIP5 models: ACCESS1-0, ACCESS1-3, GFDL-CM3, MPI-ESM-LR, MPI-ESM-MR, MPI-ESM-P.
- 21 CMIP6 models: ACCESS-CM2, ACCESS-ESM1-5, AWI-CM-1-1-MR, CAMS-CSM1-0, CanESM5, CESM2, CESM2-WACCM, FGOALS-f3-L, GISS-E2-1-G, GISS-E2-1-H, HadGEM3-GC31-LL, HadGEM3-GC31-MM, INM-CM4-8, INM-CM5-0, MPI-ESM1-2-HR, MPI-ESM1-2-LR, MRI-ESM2-0, NESM3, NorESM2-LM, NorESM2-MM, UKESM1-0-LL.

Analyses equivalent to the one shown in Fig. 2 but for other choices of percentage of observed variance thresholds (0%, 50%, 80%, 90%) are provided in Supplementary Fig. 16.

For a few of the selected models, the relevant SWOOSH period (January 1990 to December 2020) could not be matched with a consistent set of simulations. Instead, we sampled equivalent months from their historical simulations only. For CMIP5, this concerns MPI-ESM-P for which we considered the period 1968–2004, amounting to the same number of samples (note that, for example, the period 1984 to 1990 is excluded according to the data mask derived from SWOOSH). For CMIP6, the following selected models are affected: CESM2, FGOALS-f3-L, GISS-E2-1-H, HadGEM3-GC31-LL, HadGEM3-GC31-MM, INM-CM4-8, INM-CM5-0, MPI-ESM1-2-HR, MPI-ESM1-2-LR, NESM3, NorESM2-LM, NorESM2-MM. For these CMIP6 models, we instead used data covering the period 1977–2013. To keep consistency with the SWOOSH record as close as possible, we applied the mask representing SWOOSH data gaps to each model dataset from 1984 onwards, which includes masking of the period immediately following the Mt. Pinatubo eruption in 1991, which could otherwise have been considered an unusual event in the CMIP data not characterized by SWOOSH^42^.

### Statistical-learning framework

For each CMIP model and SWOOSH/reanalysis pair of specific humidity and temperature data, we train a predictive function *f* (see equation (1)). The exclusion of lags or the addition of time lags longer than ${\tau }_{\max }=2$ months do not further improve the performance (Extended Data Fig. 1). To quasi-linearize the *T*–*q*_strat_ relationships, we apply the natural logarithm to the specific humidity data, which also improves the overall predictive performance of the learned functions, in particular, under extrapolation (Extended Data Fig. 1c).

Here we use temperatures within 60° N–60° S at each of the five atmospheric pressure levels as predictors. Our set-up is constrained by our empirical results that extending the area of predictors to the polar regions did neither improve the predictive performance on historical test data nor on the abrupt-4 × CO_2_ simulations (Extended Data Fig. 1d,e). However, in particular for observations, we received the best cross-validation results on historical data when using 60° N–60° S instead of only tropical (30° N–30° S) temperatures. In a classic statistical-learning set-up of training, cross-validation and separate testing, we therefore chose the best performing configuration for the historical cross-validation data also for the abrupt-4 × CO_2_ ‘test’ scenario. We also explored the sensitivity of the extrapolation results to a longer training period (Extended Data Fig. 1f) and to the number of pressure levels at which temperature is considered as predictor (seven/three/one in Extended Data Fig. 1g,h,i). As another simplification and to speed up the learning process, we interpolated the temperature data for each CMIP model and reanalysis dataset to a common 5° × 5° (latitude × longitude) grid. This coarser spatial resolution also allows us to homogenize the predictor resolution for all temperature datasets, which is necessary to later combine different sets of temperature predictors and ridge coefficients **Θ** for the observational constraint.

To estimate the coefficients **Θ**, we use ridge regression^40^, which here minimizes the cost function

2 $${J}_{{{{\rm{ridge}}}}}({{{\boldsymbol{\Theta }}}})=\mathop{\sum}\limits_{t}{\left(\log \left({q}_{{{{\rm{strat}}}},t}\right)-\mathop{\sum}\limits_{{{{{i}}}},{{{{j}}}},{{{{k}}}},\tau }{{{\Theta }}}_{{{{{ijk}}}},\tau }{{{\rm{d}}}}{T}_{{{{{ijk}}}}}(t-\tau )\right)}^{2}+\,\alpha \mathop{\sum}\limits_{{{{{i}}}},{{{{j}}}},{{{{k}}}},\tau }{{{\Theta }}}_{{{{{ijk}}}},\tau }^{2}$$

over 315 monthly mean samples indexed by *t*. The total number *M* of temperature predictors is 25,920 (5 levels × 24 latitudes × 72 longitudes × 3 months for maximum lag ${\tau }_{\max }=2$). This large number of predictors, especially given the limited length of the observational record, would lead to overfitting using multiple linear regression (MLR). Next, to avoiding overfitting, ridge regression is also known for its good performance in managing ill-posed problems with many collinear predictors^41,57^. Note that the first term in equation (2) is the MLR least squares error, which, as discussed, tends to overfit the data given large *M*. Ridge regression addresses overfitting through the second *l*^2^-norm regularization term, which penalizes large absolute values for **Θ**, modulated by the choice for the regularization parameter *α*. To approximate optimal *α*, we use fivefold cross-validation searching over *α* ∈ [0.0001, 0.0003, 0.1, …, 1 × 10^9^] and evaluate according to the *r*^2^ scores (coefficients of determination; ref. ^58^ provides a detailed explanation) as defined by Python’s scikit-learn package^59^ across the historical validation sets. This general search range for *α* was determined incrementally following tests showing that larger and smaller values for *α* would never be selected during cross validation. As mentioned above, we standardize temperature time series at each grid point to zero mean and unit standard deviation (over the historical period) to ensure that they are considered equally and so that the absolute magnitudes of the resulting sensitivities are reflective of their relative physical importance^57^. When combining **Θ** derived from SWOOSH/reanalysis pairs with CMIP temperature responses under 4 × CO_2_, we therefore re-scale the temperature fields according to the grid point *σ* values of the reanalysis dataset to represent the relative amplitude of the CMIP modelled temperature anomalies consistently. Due to the standard scaling of temperatures and our focus on the SWV response per degree warming, we do not carry over baseline model biases in mean values of temperature and humidity into our observational constraint.

### Calculation of framework-related uncertainty

We follow a similar approach to Ceppi and Nowack^41^, in which the uncertainty in the constraint is calculated in several steps. First, we obtain a probability distribution of the observational prediction (*x* axis of Fig. 2a; solid red curve) by combining the uncertainties in **Θ**_obs_, denoted *σ*_Θ_, with those due to the different CMIP 4 × CO_2_ temperature responses, *σ*_T_. For this, we first linearly combine all of the 150 estimates of **Θ**_obs_ with each of the 27 CMIP ${{\mathbf{T}}}_{4\,\times\,{{{{\rm{CO}}}}}_{2}}$ fields, leading to 4,050 observationally constrained *T*_g_-normalized *q*_strat_ predictions. To obtain *σ*_Θ_, we first take the multi-model mean over all predictions made using the same set of observed coefficients and subsequently calculate the standard deviation of these 150 samples. We follow the same procedure for *σ*_T_ but now averaging estimates involving the same UTLS temperature response, calculating the standard deviation of the resulting 27 estimates. These uncertainties are then combined in quadrature, ${\sigma }_{p}=\sqrt{{\sigma }_{{{\Theta }}}\!^{2}+{\sigma }_{{{{\rm{T}}}}}\!^{2}}$, to yield the uncertainty for the observational prediction *q*_strat,p_.

Next, this observational prediction uncertainty is convolved with the prediction error, calculated via standard least squares regression formulas^60^, whose 5–95% interval are represented by dashed red curves in Fig. 2a. This yields a probability distribution for the actual normalized *q*_strat_ response *q*_strat,a_ on the *y* axis of Fig. 2a:

3 $$P({{{{{q}}}}}_{{{{\rm{strat}}}},{{{\rm{a}}}}})=\int\nolimits_{-\infty }^{+\infty }P({{{{{q}}}}}_{{{{\rm{strat}}}},{{{\rm{a}}}}}| {{{{{q}}}}}_{{{{\rm{strat}}}},{{{\rm{p}}}}})P({{{{{q}}}}}_{{{{\rm{strat}}}},{{{\rm{p}}}}})\ {{{\rm{d}}}}{{{{{q}}}}}_{{{{\rm{strat}}}},{{{\rm{p}}}}}$$

where the conditional probability *P*(*q*_strat,a_∣*q*_strat,p_) represents the prediction error. *P*(*q*_strat,a_) is calculated numerically by Monte Carlo sampling, with a sample size of 10^7^, and we apply a Gaussian kernel smoother to the result with a standard deviation of 0.01 ppmv K^−1^ to obtain the final probability distribution.

### Stratospheric water vapour feedback calculation

We approximate the implications of our tropical lower SWV constraint for also constraining the overall SWV radiative climate-feedback parameter^20,46^. This is justified by our empirical finding that the *q*_strat_ metric is highly correlated with SWV feedback parameters estimated from radiative transfer calculations (Pearson’s *r* = 0.85; Extended Data Fig. 3). For this purpose, we combined the feedback parameters for the six selected CMIP5 models calculated by Banerjee et al.^20^ and ran additional calculations for the 21 selected CMIP6 models. We then followed the same regression approach as taken for the observational constraint in Fig. 2a but replacing the variable along the *y* axis by the SWV feedback parameters. With this procedure, we obtain an observationally constrained 90% confidence interval for the feedback parameter of 0.086–0.201 W m^−2^ K^−1^, equalling an uncertainty reduction of 0.05 W m^−2^ K^−1^ over the 90% confidence interval (0.091–0.256 W m^−2^ K^−1^) for the 27 CMIP models. We computed the SWV feedback for the CMIP6 models using the Parallel Offline Radiative Transfer programme^61^ and following the procedure outlined in Banerjee et al. Briefly, for each model we computed the SWV change between the abrupt-4 × CO_2_ and pre-industrial control simulations, based on the last 50 years of each simulation. We input these water vapour fields into the Parallel Offline Radiative Transfer programme to compute the stratospherically adjusted net tropopause radiative flux change for each model individually using the fixed dynamical heating approximation. Then, the SWV feedback (in W m^−2^ K^−1^) is computed by dividing the tropopause radiative flux change by the global mean surface temperature change (again, averaged over the last 50 years of each simulation).

## Online content

Any methods, additional references, Nature Portfolio reporting summaries, source data, extended data, supplementary information, acknowledgements, peer review information; details of author contributions and competing interests; and statements of data and code availability are available at 10.1038/s41561-023-01183-6.

## Supplementary information

Supplementary Information Supplementary Tables 1–3 and Figs. 1–16.

## Data Availability

All observational, reanalysis and climate model datasets used in this study are publicly available. SWOOSH data can be found at https://csl.noaa.gov/groups/csl8/swoosh/. CMIP data were obtained from the UK Center for Environmental Data Analysis portal (https://esgf-index1.ceda.ac.uk/search/cmip6-ceda/). MERRA-2 data were obtained from the Collaborative REAnalysis Technical Environment (CRE-ATE) project (https://esgf-node.llnl.gov/search/create-ip/). JRA-55 data were downloaded from the National Center for Atmospheric Research/University Corporation for Atmospheric Research Research Data Archive (https://rda.ucar.edu/datasets/ds628.1/). ERA5 data were downloaded from the Copernicus Climate Data Store (10.24381/cds.f17050d7). In addition, pre-processed versions of the data used to run the calculations and source data to produce the figures in the manuscript are archived on figshare (10.6084/m9.figshare.22335712).

## Code availability

Python Jupyter notebooks used for the data analysis and production of figures are available on the Github webpage of P.N. at https://github.com/peernow/SWV_Nature_Geoscience.
