# Supplementary material for: Response of stratospheric water vapour to warming constrained by satellite observations
Source: Nat Geosci. 2023 Jun 26;16(7):577–83. doi: 10.1038/s41561-023-01183-6 (PMC10333120; doi:10.1038/s41561-023-01183-6)
Supplement: Supplementary file 1 — Supplementary Tables 1–3 and Figs. 1–16. [file 41561_2023_1183_MOESM1_ESM.pdf]

nature geoscience

Article https://doi.org/10.1038/s41561-023-01183-6

Response of stratospheric water vapour to warming constrained by satellite observations

In the format provided by the authors and unedited

Supplementary Material | Table of Contents

This file contains:

- Supplementary Tables 1–3, summarizing CMIP radiative transfer calculations and q_{strat} responses; the latter normalized by global mean surface temperature change or tropical upper tropospheric warming.
- Supplementary Figs. 1–2, showing SWOOSH/CMIP q_{strat} anomalies for the ridge regression training period.
- Supplementary Fig. 3, comparing ridge regression and index-based MLR 4xCO_2 predictions.
- Supplementary Figs. 4–7, comparing ridge predictions against actual 4xCO_2 simulation results.
- Supplementary Fig. 8, providing constraints for q_{strat} normalized by tropical upper tropospheric warming.
- Supplementary Figs. 9–11, showing ridge coefficients for simultaneous and lagged temperature predictors.
- Supplementary Figs. 12–15, showing latitude-height cross sections of ridge coefficients.
- Supplementary Fig. 16, showing 4xCO_2 results depending on the variance threshold set for model selection.

Model name	LW [W/m2]	SW [W/m2]	net [W/m2]	Delta T (K)	Feedback [W/m2/K]
ACCESS-CM2	1.8783	-0.2790	1.5994	6.3478	0.2520
ACCESS-ESM1-5	0.9718	-0.1206	0.8512	5.3827	0.1581
AWI-CM-1-1-MR	1.0969	-0.1143	0.9826	5.4243	0.1812
CAMS-CSM1-0	0.9847	-0.1192	0.8655	4.0918	0.2115
CanESM5	1.7210	-0.1400	1.5810	8.0084	0.1974
CESM2	2.7290	-0.2907	2.4384	11.2143	0.2174
CESM2-WACCM	1.0841	-0.0947	0.9894	6.3681	0.1554
FGOALS-f3-L	1.0535	-0.1331	0.9204	5.0090	0.1837
GISS-E2-1-G	0.6644	-0.0683	0.5961	4.1467	0.1438
GISS-E2-1-H	0.7332	-0.0749	0.6582	4.9069	0.1341
HadGEM3-GC31-LL	2.2094	-0.3408	1.8685	7.2311	0.2584
HadGEM3-GC31-MM	2.2345	-0.3413	1.8932	7.2507	0.2611
INM-CM4-8	0.2469	0.0122	0.2590	3.5887	0.0722
INM-CM5-0	0.2571	-0.0042	0.2530	3.5371	0.0715
MPI-ESM1-2-HR	0.9968	-0.1030	0.8937	4.8516	0.1842
MPI-ESM1-2-LR	1.0122	-0.1063	0.9060	4.9259	0.1839
MRI-ESM2-0	0.7356	-0.0606	0.6751	4.6884	0.1440
NESM3	1.6505	-0.1920	1.4585	6.9447	0.2100
NorESM2-LM	1.1592	-0.1166	1.0426	6.6166	0.1576
NorESM2-MM	0.7591	-0.0831	0.6760	3.9670	0.1704
UKESM1-0-LL	1.7749	-0.2513	1.5236	7.7075	0.1977

Supplementary Table 1 | Main results of the CMIP6 radiative transfer calculations. From left to right values for: adjusted tropopause longwave (LW) flux, adjusted tropopause shortwave (SW) flux, net tropopause radiative flux, temperature difference 4xCO_2 to pre-industrial simulations, and the estimated SWV feedback parameters.

CMIP5/6 model name	Normalized q_{strat} response (ppmv K^{-1})	Reference paper
ACCESS1-0	0.77	Bi et al. (2013); Dix et al. (2013) (1; 2)
ACCESS1-3	0.34	Bi et al. (2013); Dix et al. (2013) (1; 2)
ACCESS-CM2	1.24	Bi et al. (2020) (3)
ACCESS-ESM1-5	0.34	Ziehn et al. (2020) (4)
AWI-CM-1-1-MR	0.60	Semmler et al. (2020) (5)
BCC-CSM1-1	0.40	Xin et al. (2013); Wu et al (2014) (6; 7)
BCC-CSM1-1-m	0.40	Xin et al. (2013); Wu et al (2014) (6; 7)
BCC-CSM2-MR	0.53	Wu et al. (2019) (8)
BCC-ESM1	0.48	Wu et al. (2020) (9)
BNU-ESM	0.37	Ji et al. (2014) (10)
CAMS-CSM1-0	1.09	Rong et al. (2019) (11)
CanESM2	0.40	Von Salzen et al. (2013) (12)
CanESM5	0.98	Swart et al. (2019) (13)
CCSM4	0.31	Gent et al. (2011) (14)
CESM2	0.56	Danabasoglu et al. (2020) (15)
CESM2-WACCM	0.28	Danabasoglu et al. (2020) (15)
CNRM-CM5	1.17	Voldoire et al. (2013) (16)
CNRM-CM6-1	0.18	Voldoire et al. (2019) (17)
CNRM-ESM2-1	0.29	Seferian et al. (2019) (18)
CSIRO-Mk3-6-0	2.24	Jeffrey et al. (2013) (19)
E3SM-1-0	0.49	Golaz et al. (2019) (20)
EC-Earth3-Veg	0.50	Döescher et al. (2022) (21)
EC-EARTH	0.53	Hazeleger et al. (2012) (22)
FGOALS-f3-L	0.46	He et al. (2019) (23)
FGOALS-g2	0.36	Li et al. (2013) (24)
FGOALS-g3	0.51	Pu et al. (2020) (25)
GFDL-CM3	0.52	Griffies et al. (2011) (26)
GFDL-CM4	0.43	Held et al. (2019) (27)
GFDL-ESM2G	1.43	Dunne et al. (2012) (28)
GFDL-ESM2M	1.57	Dunne et al. (2012) (28)
GFDL-ESM4	0.28	Dunne et al. (2020) (29)
GISS-E2-1-G	0.68	Kelley et al. (2020) (30)
GISS-E2-1-H	0.59	Kelley et al. (2020) (30)
GISS-E2-H	0.55	Schmidt et al. (2014) (31)
GISS-E2-R	0.58	Schmidt et al. (2014) (31)
HadGEM2-ES	0.73	Jones et al. (2011) (32)
HadGEM3-GC31-LL	1.37	Andrews et al. (2020) (33)
HadGEM3-GC31-MM	1.41	Andrews et al. (2020) (33)
INM-CM4	0.16	Volodin et al. (2010) (34)
INM-CM4-8	0.18	Volodin et al. (2018)a (35)
INM-CM5-0	0.08	Volodin et al. (2018)b (36)
IPSL-CM5A-MR	1.07	Dufresne et al. (2013) (37)
IPSL-CM5B-LR	0.25	Dufresne et al. (2013) (37)
IPSL-CM6A-LR	0.62	Boucher et al. (2020) (38)
MIROC5	0.26	Watanabe et al. (2010) (39)
MIROC6	0.24	Tatebe et al. (2019) (40)
MIROC-ES2L	0.24	Hajima et al. (2020) (41)
MIROC-ESM	0.55	Watanabe et al. (2011) (42)
MPI-ESM1-2-HR	0.55	Gutjahr et al. (2019); Müller et al. (2018) (43; 44)
MPI-ESM1-2-LR	0.65	Mauritsen et al. (2019) (45)
MPI-ESM-LR	0.63	Giorgetta et al. (2013) (46)
MPI-ESM-MR	0.53	Giorgetta et al. (2013) (46)
MPI-ESM-P	0.63	Giorgetta et al. (2013) (46)
MRI-CGCM3	0.90	Yukimoto et al. (2012) (47)
MRI-ESM2-0	0.22	Yukimoto et al. (2019) (48)
NESM3	0.94	Cao et al. (2018) (49)
NorESM1-M	0.29	Iversen et al. (2013) (50)
NorESM2-LM	0.24	Seland et al. (2020) (51)
NorESM2-MM	0.34	Seland et al. (2020) (51)
SAM0-UNICON	0.44	Park et al. (2019) (52)
UKESM1-0-LL	0.74	Sellar et al. (2020) (53)

Supplementary Table 2 | Estimates of 70 hPa zonal mean tropical (30°N–30°S) specific humidity responses per degree global warming (ppmv K^{-1}). For all 61 CMIP5 and CMIP6 models considered here, estimated from linear regressions applied to annual mean data from abrupt-4xCO_2 simulations. The third column provides the CMIP5/CMIP6 model reference paper (to the best of the authors' awareness).

CMIP5/6 model name	Normalized q_{strat} response (ppmv K^{-1})	Reference paper
ACCESS1-0	1.11	Bi et al. (2013); Dix et al. (2013) (1; 2)
ACCESS1-3	0.32	Bi et al. (2013); Dix et al. (2013) (1; 2)
ACCESS-CM2	0.93	Bi et al. (2020) (3)
ACCESS-ESM1-5	0.35	Ziehn et al. (2020) (4)
AWI-CM-1-1-MR	0.47	Semmler et al. (2020) (5)
BCC-CSM1-1	0.46	Xin et al. (2013); Wu et al (2014) (6; 7)
BCC-CSM1-1-m	0.36	Xin et al. (2013); Wu et al (2014) (6; 7)
BCC-CSM2-MR	0.51	Wu et al. (2019) (8)
BCC-ESM1	0.45	Wu et al. (2020) (9)
BNU-ESM	0.50	Ji et al. (2014) (10)
CAMS-CSM1-0	0.79	Rong et al. (2019) (11)
CanESM2	0.37	Von Salzen et al. (2013) (12)
CanESM5	0.67	Swart et al. (2019) (13)
CCSM4	0.37	Gent et al. (2011) (14)
CESM2	0.47	Danabasoglu et al. (2020) (15)
CESM2-WACCM	0.26	Danabasoglu et al. (2020) (15)
CNRM-CM5	2.25	Voldoire et al. (2013) (16)
CNRM-CM6-1	0.28	Voldoire et al. (2019) (17)
CNRM-ESM2-1	0.36	Seferian et al. (2019) (18)
CSIRO-Mk3-6-0	1.89	Jeffrey et al. (2013) (19)
E3SM-1-0	0.48	Golaz et al. (2019) (20)
EC-Earth3-Veg	0.50	Döescher et al. (2022) (21)
EC-EARTH	0.80	Hazeleger et al. (2012) (22)
FGOALS-f3-L	0.64	He et al. (2019) (23)
FGOALS-g2	0.51	Li et al. (2013) (24)
FGOALS-g3	0.55	Pu et al. (2020) (25)
GFDL-CM3	0.47	Griffies et al. (2011) (26)
GFDL-CM4	0.50	Held et al. (2019) (27)
GFDL-ESM2G	1.33	Dunne et al. (2012) (28)
GFDL-ESM2M	1.45	Dunne et al. (2012) (28)
GFDL-ESM4	0.29	Dunne et al. (2020) (29)
GISS-E2-1-G	0.56	Kelley et al. (2020) (30)
GISS-E2-1-H	0.58	Kelley et al. (2020) (30)
GISS-E2-H	0.62	Schmidt et al. (2014) (31)
GISS-E2-R	0.64	Schmidt et al. (2014) (31)
HadGEM2-ES	1.07	Jones et al. (2011) (32)
HadGEM3-GC31-LL	1.06	Andrews et al. (2020) (33)
HadGEM3-GC31-MM	1.09	Andrews et al. (2020) (33)
INM-CM4	0.33	Volodin et al. (2010) (34)
INM-CM4-8	0.38	Volodin et al. (2018)a (35)
INM-CM5-0	0.24	Volodin et al. (2018)b (36)
IPSL-CM5A-MR	0.67	Dufresne et al. (2013) (37)
IPSL-CM5B-LR	0.46	Dufresne et al. (2013) (37)
IPSL-CM6A-LR	0.48	Boucher et al. (2020) (38)
MIROC5	0.27	Watanabe et al. (2010) (39)
MIROC6	0.20	Tatebe et al. (2019) (40)
MIROC-ES2L	0.23	Hajima et al. (2020) (41)
MIROC-ESM	0.52	Watanabe et al. (2011) (42)
MPI-ESM1-2-HR	0.43	Gutjahr et al. (2019); Müller et al. (2018) (43; 44)
MPI-ESM1-2-LR	0.46	Mauritsen et al. (2019) (45)
MPI-ESM-LR	0.48	Giorgetta et al. (2013) (46)
MPI-ESM-MR	0.42	Giorgetta et al. (2013) (46)
MPI-ESM-P	0.48	Giorgetta et al. (2013) (46)
MRI-CGCM3	1.24	Yukimoto et al. (2012) (47)
MRI-ESM2-0	0.29	Yukimoto et al. (2019) (48)
NESM3	0.72	Cao et al. (2018) (49)
NorESM1-M	0.39	Iversen et al. (2013) (50)
NorESM2-LM	0.30	Seland et al. (2020) (51)
NorESM2-MM	0.37	Seland et al. (2020) (51)
SAM0-UNICON	0.48	Park et al. (2019) (52)
UKESM1-0-LL	0.67	Sellar et al. (2020) (53)

Supplementary Table 3 | Estimates of 70 hPa zonal mean tropical (30°N–30°S) specific humidity responses per degree upper tropospheric warming close to the tropical cold trap (100 hPa, 20°N–20°S; ppmv K^{-1}). For all 61 CMIP5 and CMIP6 models considered here, estimated from linear regressions applied to annual mean data from abrupt-4xCO_2 simulations. The third column provides the CMIP5/CMIP6 model reference paper (to the best of the authors' awareness).

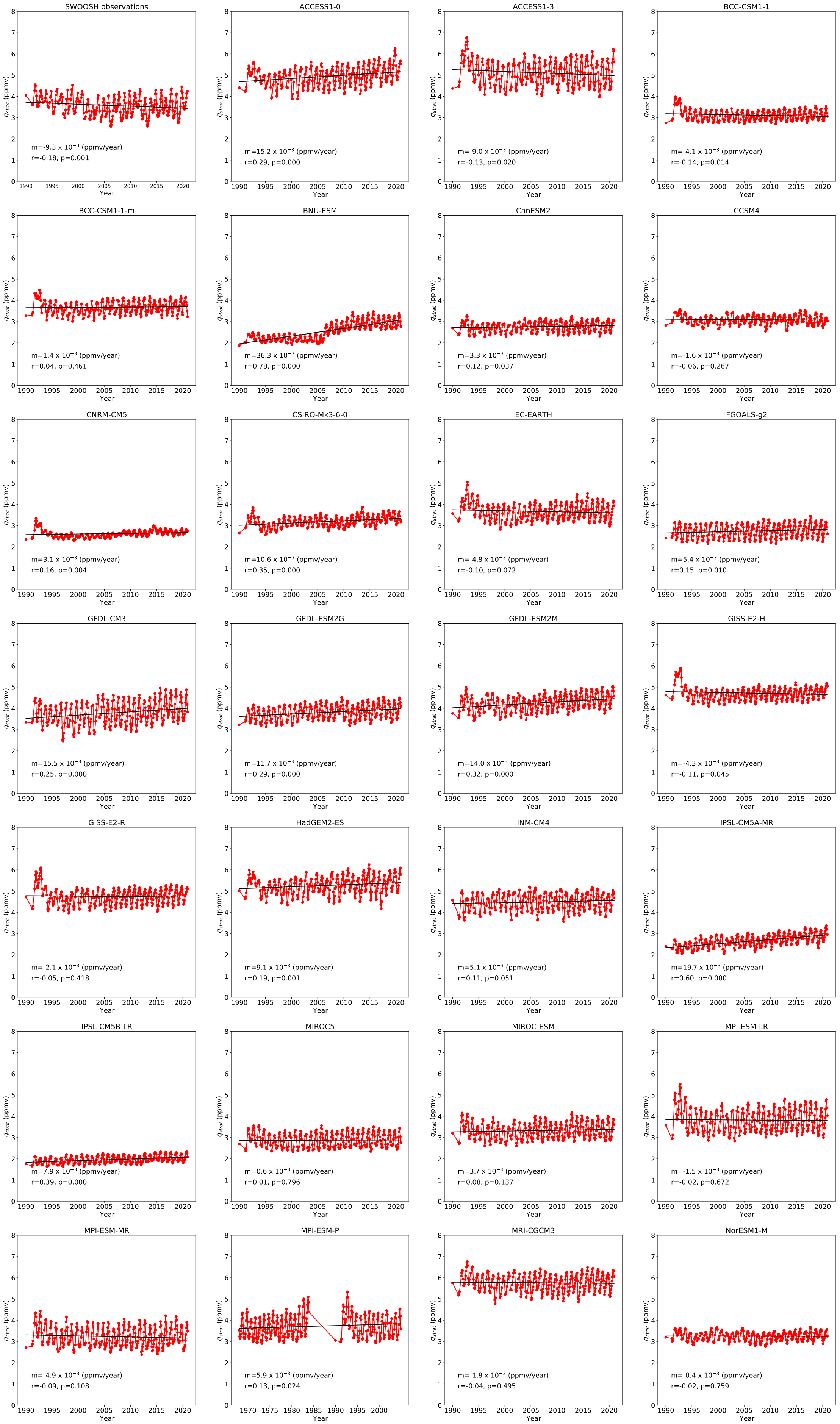

Supplementary Fig. 1 | CMIP stratospheric humidity data. Shown are 30°N–30°S spatial averages at 70 hPa for 27 CMIP5 models and SWOOSH observations (top left). All datasets are masked for missing months in SWOOSH, or similarly shifted periods (Online Methods). Black lines and inset values show the trends over time as estimated by linear regression (*m*=slope, *r*=correlation coefficient, *p*-value). **Recommendation is to zoom in to ca. 250% for pdf viewing.**

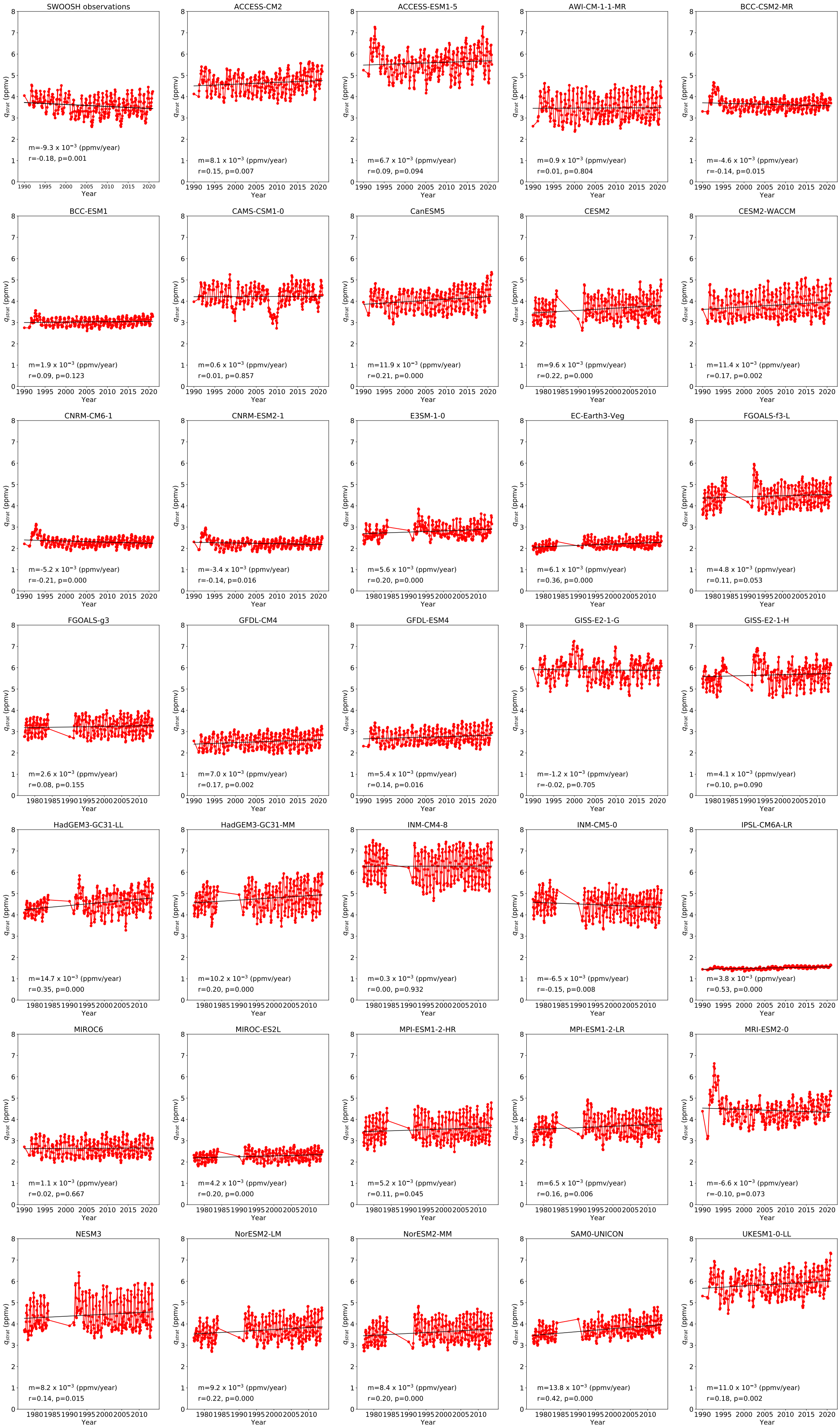

Supplementary Fig. 2 | CMIP stratospheric humidity data. Shown are 30°N–30°S spatial averages at 70 hPa for 34 CMIP6 models and SWOOSH observations (top left). All datasets are masked for missing months in SWOOSH, or similarly shifted periods (Online Methods). Black lines and inset values show the trends over time as estimated by linear regression (m=slope, r=correlation coefficient, p-value). **Recommendation is to zoom in to ca. 250% for pdf viewing.**

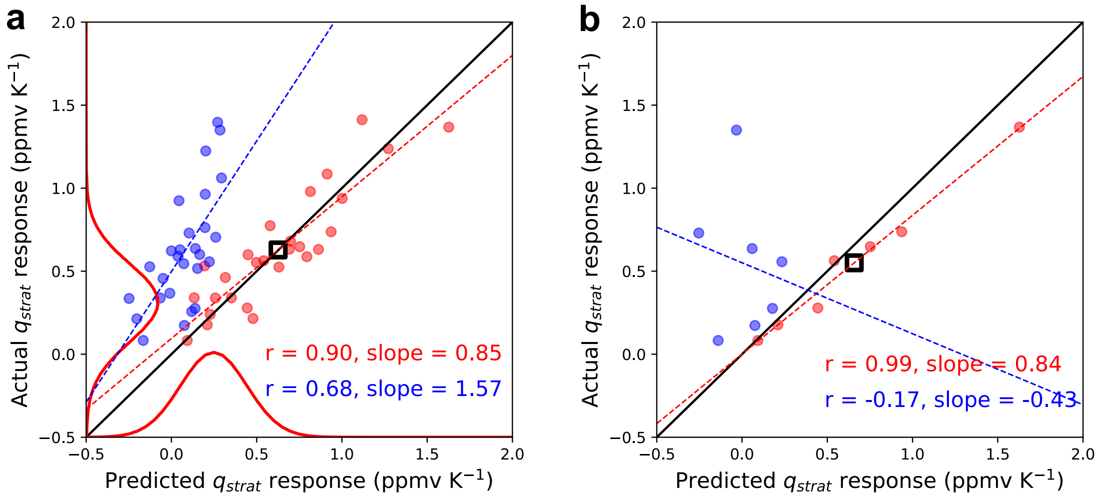

Supplementary Fig. 3 | Framework performance. As Fig. 2a in the main text (red results for ridge regressions), however, with results from climate index-based MLR regression results (blue) added. In **a**, the MLR for each CMIP model is based on monthly-mean de-seasonalized tropical (30°N–30°S) SWV anomalies at 70 hPa and a QBO-index (50 hPa, 5°N–5°S zonal winds), the NINO3.4 index (54), and zonal mean 500 hPa tropical air temperatures, following the regression approaches by Dessler et al. and Smalley et al. (55; 56; 57). Lags for the indices are included as in Smalley et al. (2017). The regressions are fit on the same historical data as the ridge regressions and are then - again as for ridge regressions in the main text - combined with the 4xCO_2 responses in those indices, normalized by each model-consistent global mean surface temperature response. In **b**, we additionally included the tropical radiative heating rate at 70 hPa as predictor (variable 'tntr' in CMIP6) as in (55; 56; 57), which was only available for a subset of seven of the models. While a good correlation between MLR-predicted and actual climate model responses is achieved in **a** (linear correlation r = 0.68), the correlation is substantially better for the ridge regressions (r = 0.90). Even more importantly, the MLR-predictions underestimate the actual 4xCO_2 responses, leading to a significant deviation from the one-to-one relationship (slope = 1.57). While the sample size of seven models in **b** is small, the extrapolation performance does not seem to be improved by additionally considering the heating rates as predictors, and that is even true on a sub-set of models that the ridge regressions apparently predict well (red circles in **b**). We also note that the heating rates should reflect changes in the Brewer-Dobson circulation, which will tend to further reduce the already underestimated SWV responses if the circulation accelerates in response to CO_2 forcing (as is typically projected by climate models; 55; 57). Overall, these results highlight the greater potential of ridge regressions to also perform better in climate forcing settings of predicting 4xCO_2 responses, which is pivotal to the performance and reliability of our observational constraint framework.

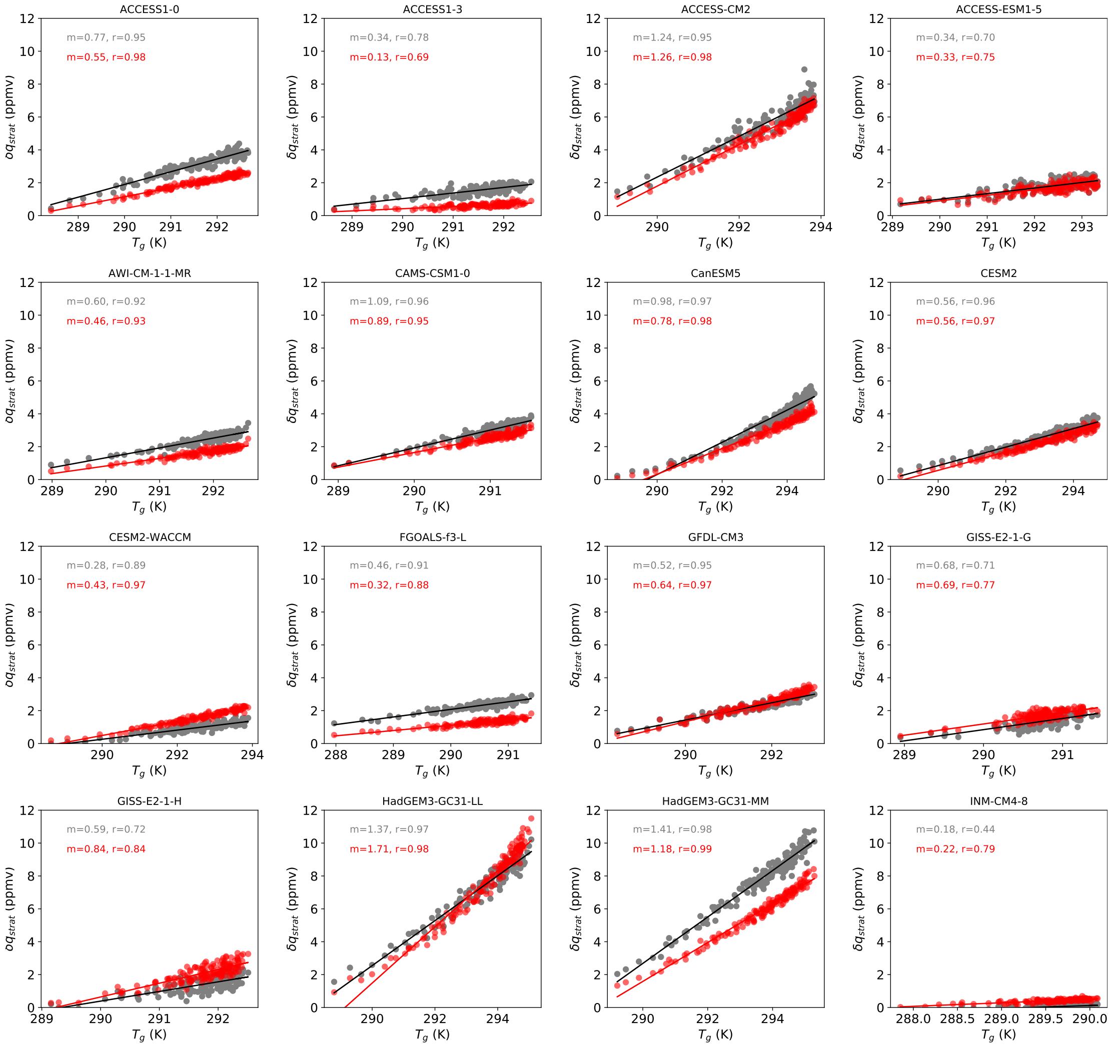

Supplementary Fig. 4 | Comparison of statistical learning predictions with actual 4xCO_2 results. Shown are results for 16 of the in total 27 CMIP models that fulfilled the minimum variance criterion. These figures follow the same logic as Figure 1d in the main text, i.e. we compare the predictions of the learned functions (red) with the actual abrupt-4xCO_2 simulation results (grey). To account for different levels of global warming simulated by each model, the change in annual mean q_{strat} is regressed against T_g, relative to the average value over the historical training period (δ). Colour-coded inset values for m represent the slope for each linear regression (in ppmv K^{-1}) and r the correlation coefficient. Note that the intercepts in each subplot can be expected to differ, because for the statistical learning predictions a historical period baseline (1990-2020) is assumed, whereas abrupt-4xCO_2 simulations are started from pre-industrial control runs (58; 59). As a result, intercept offsets will to a degree be related to modelled changes in q_{strat} from pre-industrial levels by the beginning of the 21st century under anthropogenic forcing.

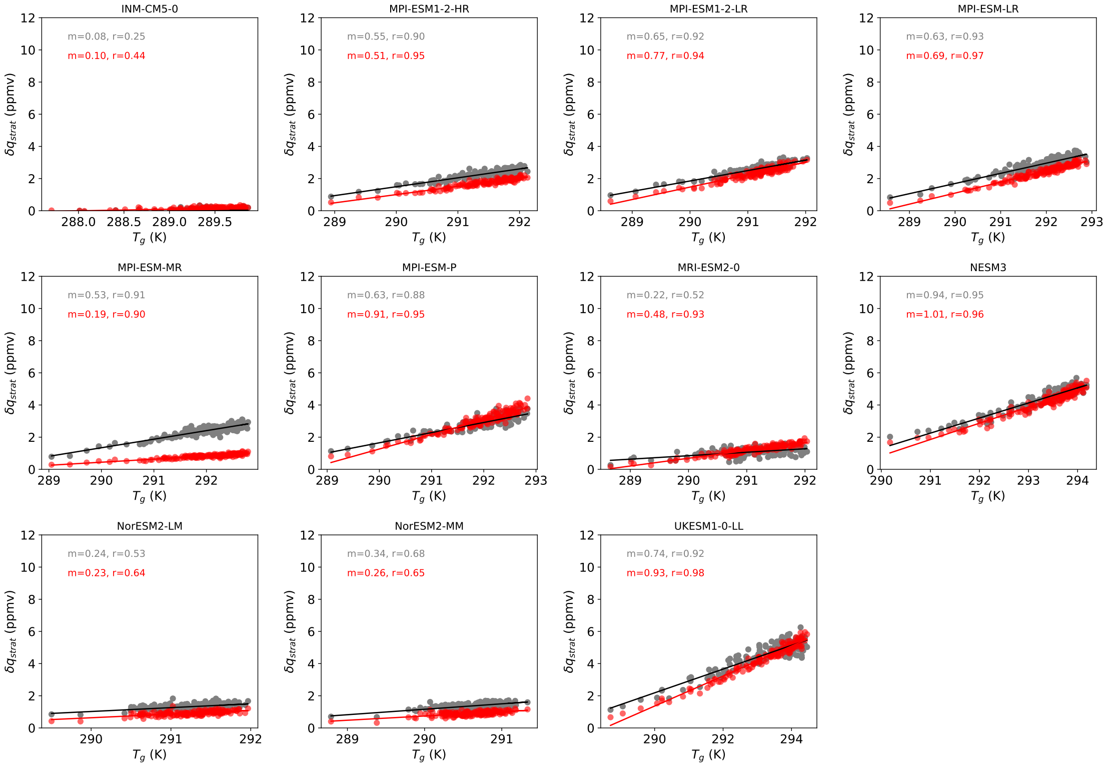

Supplementary Fig. 5 | Comparison of statistical learning predictions with actual 4xCO_2 results. Same as Supplementary Fig. 4, but for the remaining 11 CMIP5/6 models used in the main text to define the constraint.

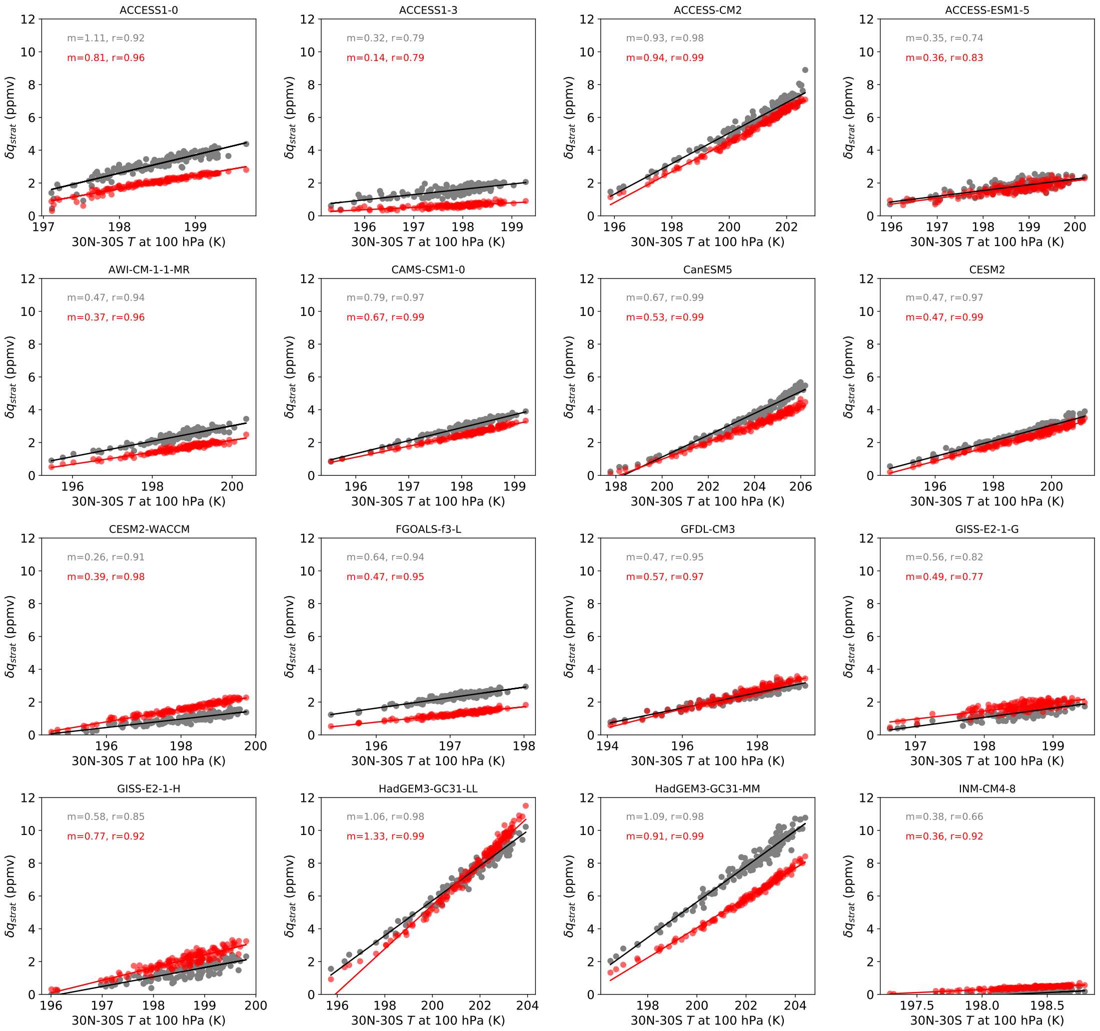

Supplementary Fig. 6 | Comparison of statistical learning predictions with actual 4xCO_2 results. Same as Supplementary Fig. 4, but normalized by upper tropospheric warming close to the tropical cold trap (100 hPa, 20°N–20°S).

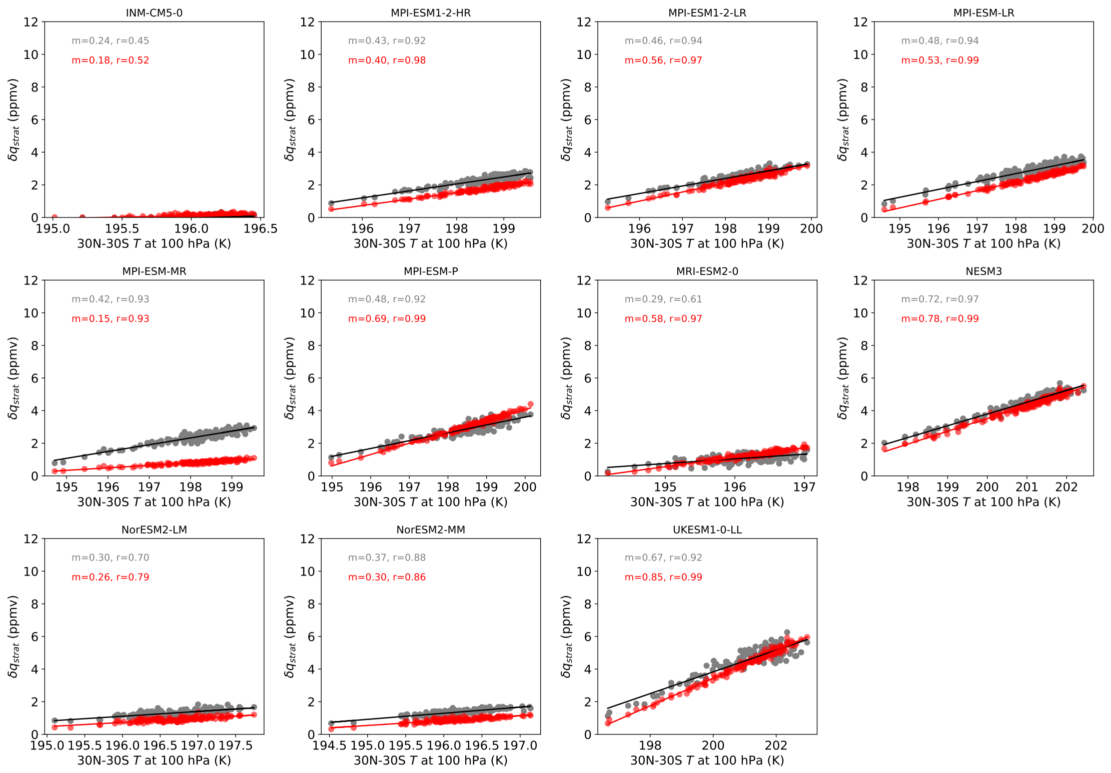

Supplementary Fig. 7 | Comparison of statistical learning predictions with actual 4xCO_2 results. Same as Supplementary Fig. 5, but normalized by upper tropospheric warming close to the tropical cold trap (100 hPa, 20°N–20°S).

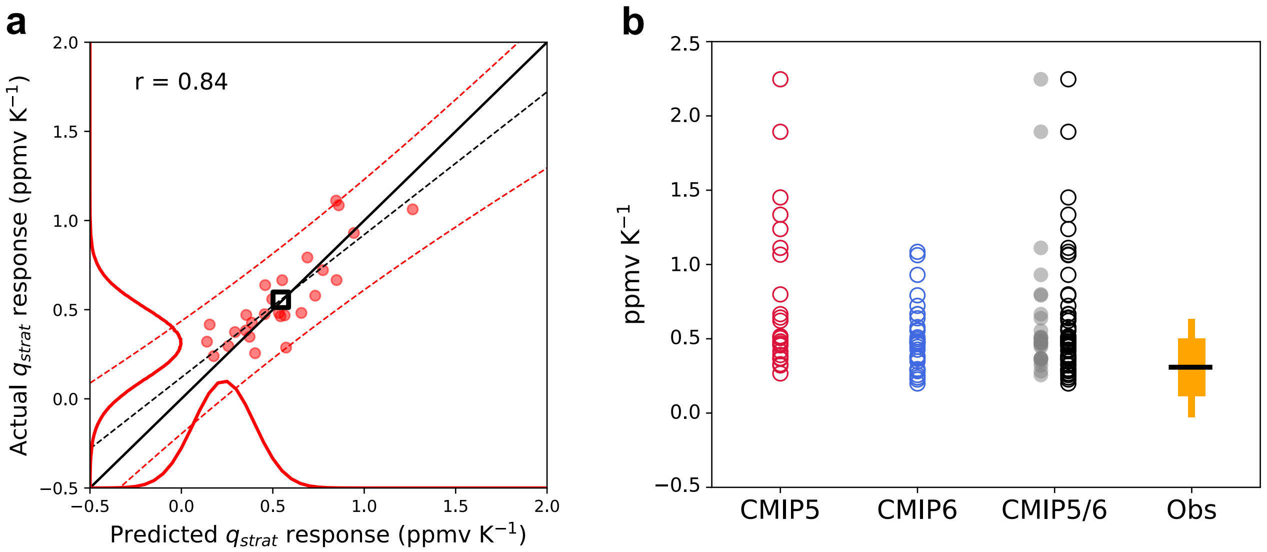

Supplementary Fig. 8 | Framework performance and the observational constraint, if normalized by 20°N-20°S zonal mean temperature at 100 hPa. **a** Red circles show abrupt-4xCO_2 simulation results ('actual') regressed against predicted changes in q_{strat}, both normalized by 20°N-20°S zonal mean temperature at 100 hPa (i.e. temperatures in proximity to the tropical tropopause), for 27 CMIP models. The multi-model-mean is indicated as a black square; the one-to-one line in solid black. Dashed lines show the least squares regression fit (black) and the 5 to 95% prediction intervals (red). The probability distributions (red curves) on the axes represent the observational estimates, with their amplitudes scaled arbitrarily. The distribution on top of the x-axis indicates the spread in normalized q_{strat} predictions based on combining functions learned from observations with the CMIP temperature responses. The final probability distribution, defining the observational constraint, is attached to the y-axis and additionally accounts for the uncertainty introduced by inaccuracies in the statistical learning predictions. **b** shows the observational constraint (n = 4050; 150 functions derived from observations × 27 CMIP temperature responses) relative to CMIP model uncertainty. Circles show the normalized changes in q_{strat} for 27 CMIP5 models (red), 34 CMIP6 models (blue), as well as their combination (black). The parallel plotted grey circles indicate the selected 27 models, which fulfill the minimum variance criterion compared to SWOOSH. These 27 models were used to validate the observational constraint framework in **a**. The observational constraint (orange) is illustrated on the right, with the horizontal black line indicating the 50th percentile (0.31 ppmv K^{-1}). The thin and thick bars denote 90% (-0.03 to 0.63 ppmv K^{-1}) and 66% (0.11 to 0.50 ppmv K^{-1}) confidence intervals, respectively. The CMIP mean (median) values are 0.73 (0.50) ppmv K^{-1} for CMIP5, 0.50 (0.48) ppmv K^{-1} for CMIP6, 0.60 (0.48) ppmv K^{-1} for the combined set of CMIP5/6, and 0.63 (0.48) ppmv K^{-1} for the 27 selected models.

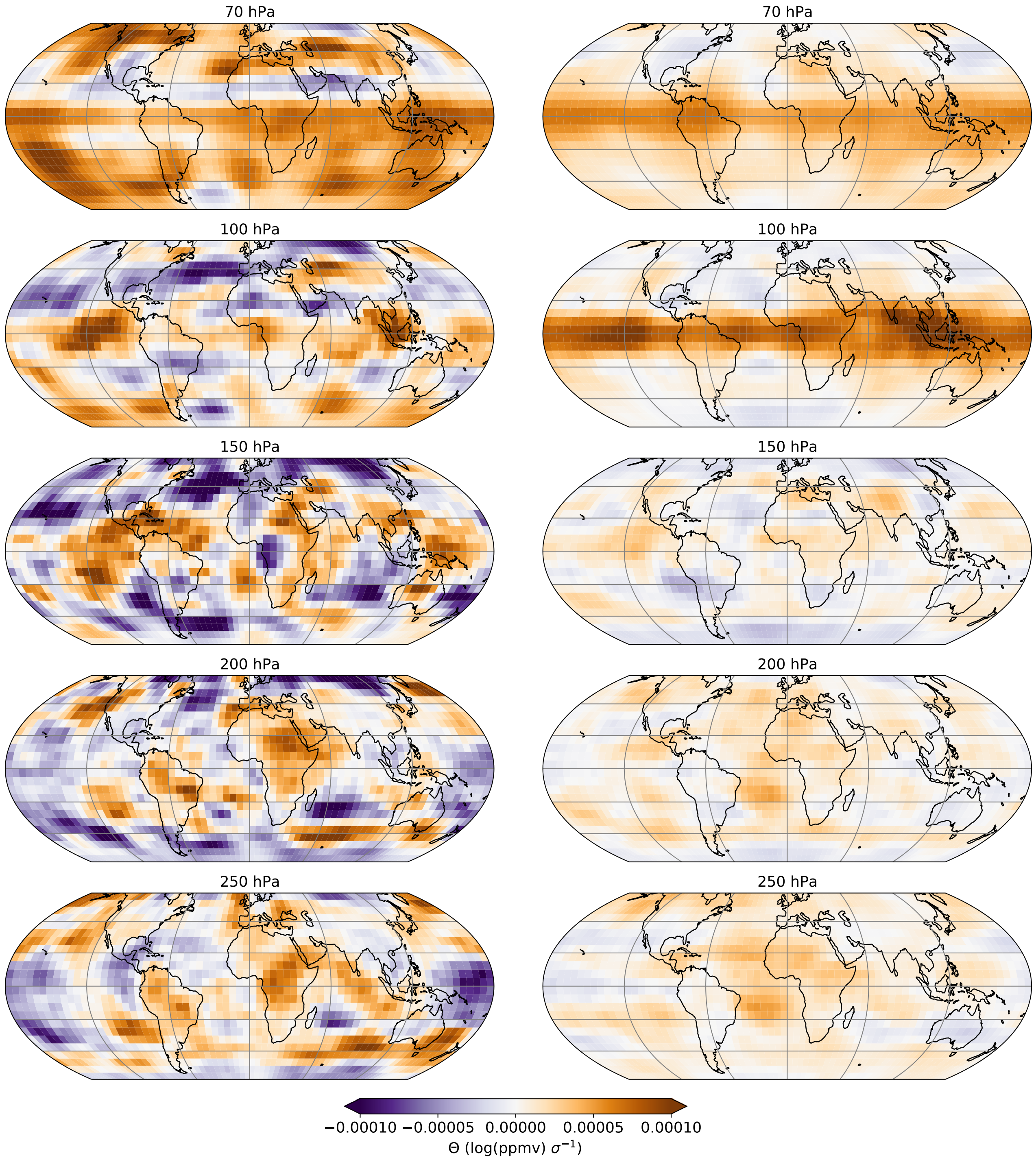

Supplementary Fig. 9 | Ridge regression coefficients Θ for τ= 2. The left column shows average coefficients across the 150 combinations of SWOOSH and reanalysis data. The right column shows equivalent results for the multi-model-mean across the 27 CMIP models. Vertical levels are as labelled at the top of each subplot. The units of the coefficients are in water vapour mixing ratio change per degree temperature change (i.e. proportional to ppmv K^{-1}); however, the temperature data has been standard-scaled (denoted by the unit σ here) for each grid cell prior to the regression. This is necessary in ridge regression to allow for a balanced consideration of each temperature grid point in the ridge regularization process (60; 61; 62). The coefficient magnitudes as shown here are therefore directly comparable, i.e. a larger magnitude negative/positive coefficient implies a greater drying/humidifying effect on the tropical lower stratosphere for a typical temperature increase in that grid box.

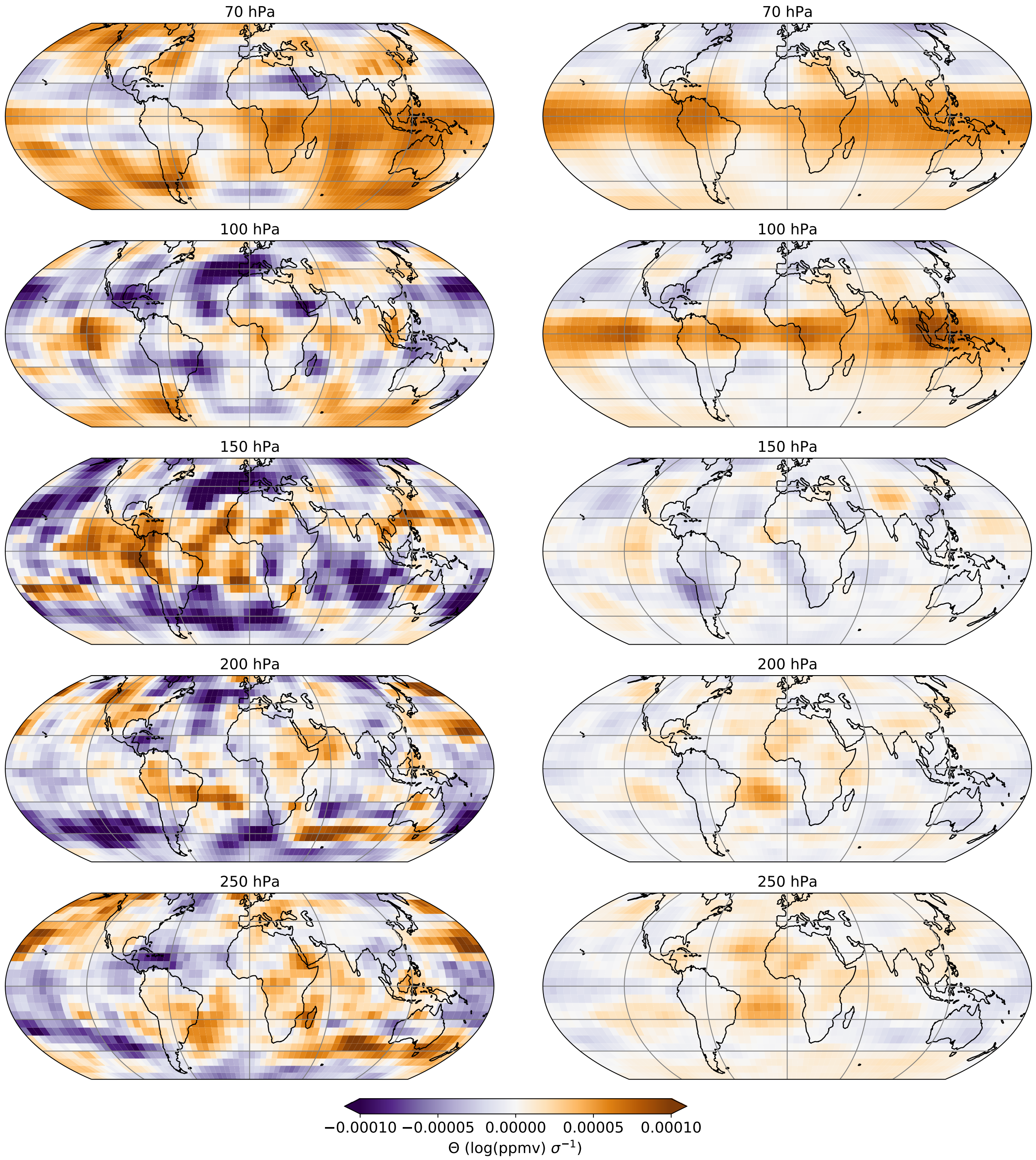

Supplementary Fig. 10 | Ridge regression coefficients Θ for τ= 1. The left column shows average coefficients across the 150 combinations of SWOOSH and reanalysis data. The right column shows equivalent results for the multi-model-mean across the 27 CMIP models. Vertical levels are as labelled at the top of each subplot. The units of the coefficients are in water vapour mixing ratio change per degree temperature change (i.e. proportional to ppmv K^{-1}); however, the temperature data has been standard-scaled (denoted by the unit σ here) for each grid cell prior to the regression. This is necessary in ridge regression to allow for a balanced consideration of each temperature grid point in the ridge regularization process (60; 61; 62). The coefficient magnitudes as shown here are therefore directly comparable, i.e. a larger magnitude negative/positive coefficient implies a greater drying/humidifying effect on the tropical lower stratosphere for a typical temperature increase in that grid box.

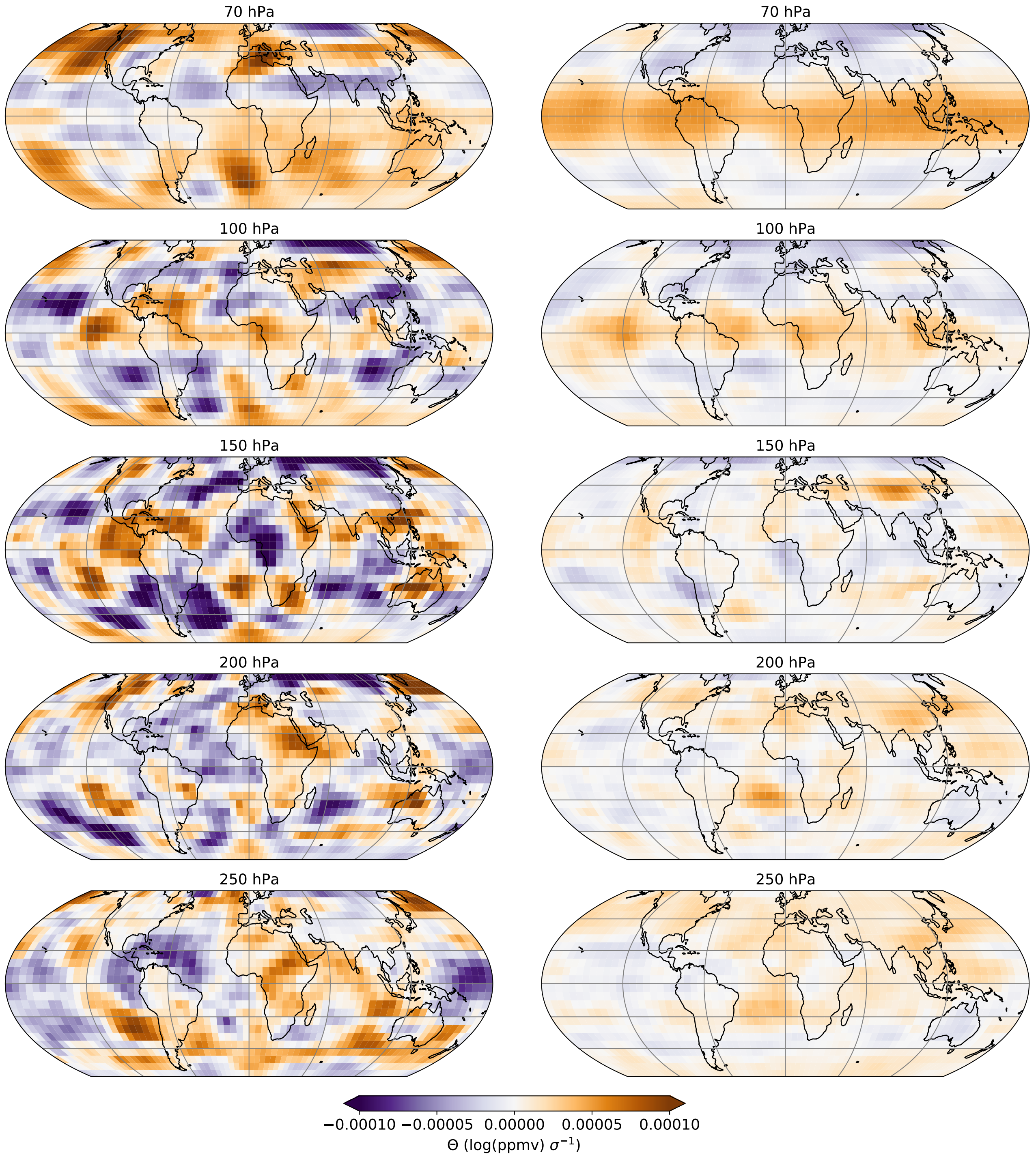

Supplementary Fig. 11 | Ridge regression coefficients Θ for τ= 0. The left column shows average coefficients across the 150 combinations of SWOOSH and reanalysis data. The right column shows equivalent results for the multi-model-mean across the 27 CMIP models. Vertical levels are as labelled at the top of each subplot. The units of the coefficients are in water vapour mixing ratio change per degree temperature change (i.e. proportional to ppmv K^{-1}); however, the temperature data has been standard-scaled (denoted by the unit σ here) for each grid cell prior to the regression. This is necessary in ridge regression to allow for a balanced consideration of each temperature grid point in the ridge regularization process (60; 61; 62). The coefficient magnitudes as shown here are therefore directly comparable, i.e. a larger magnitude negative/positive coefficient implies a greater drying/humidifying effect on the tropical lower stratosphere for a typical temperature increase in that grid box.

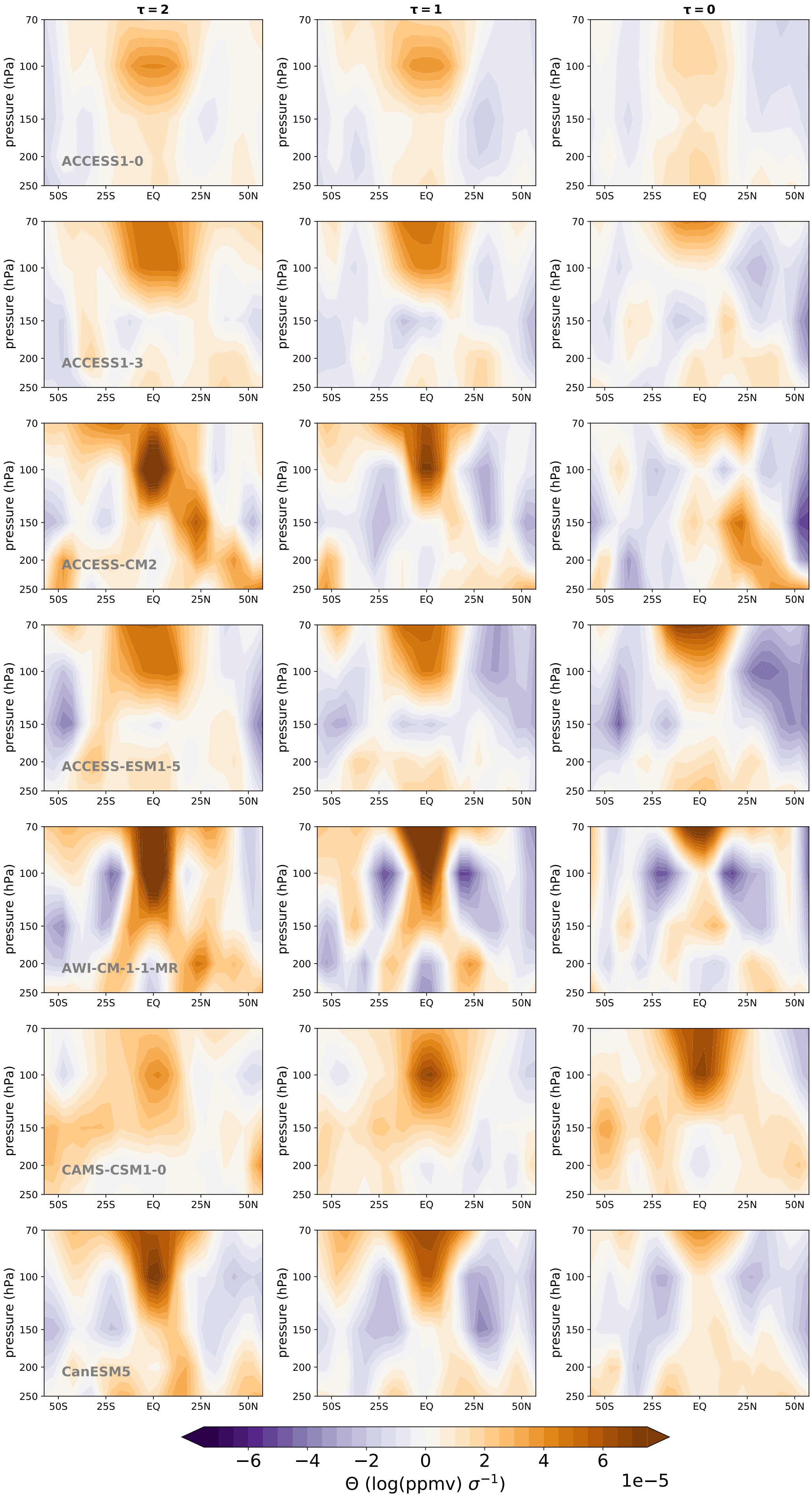

Supplementary Fig. 12 | Zonal mean cross sections of the ridge coefficients for individual CMIP models. As Fig. 4d in the main text, but for the first set of 8 of the 27 CMIP models (ordered alphabetically).

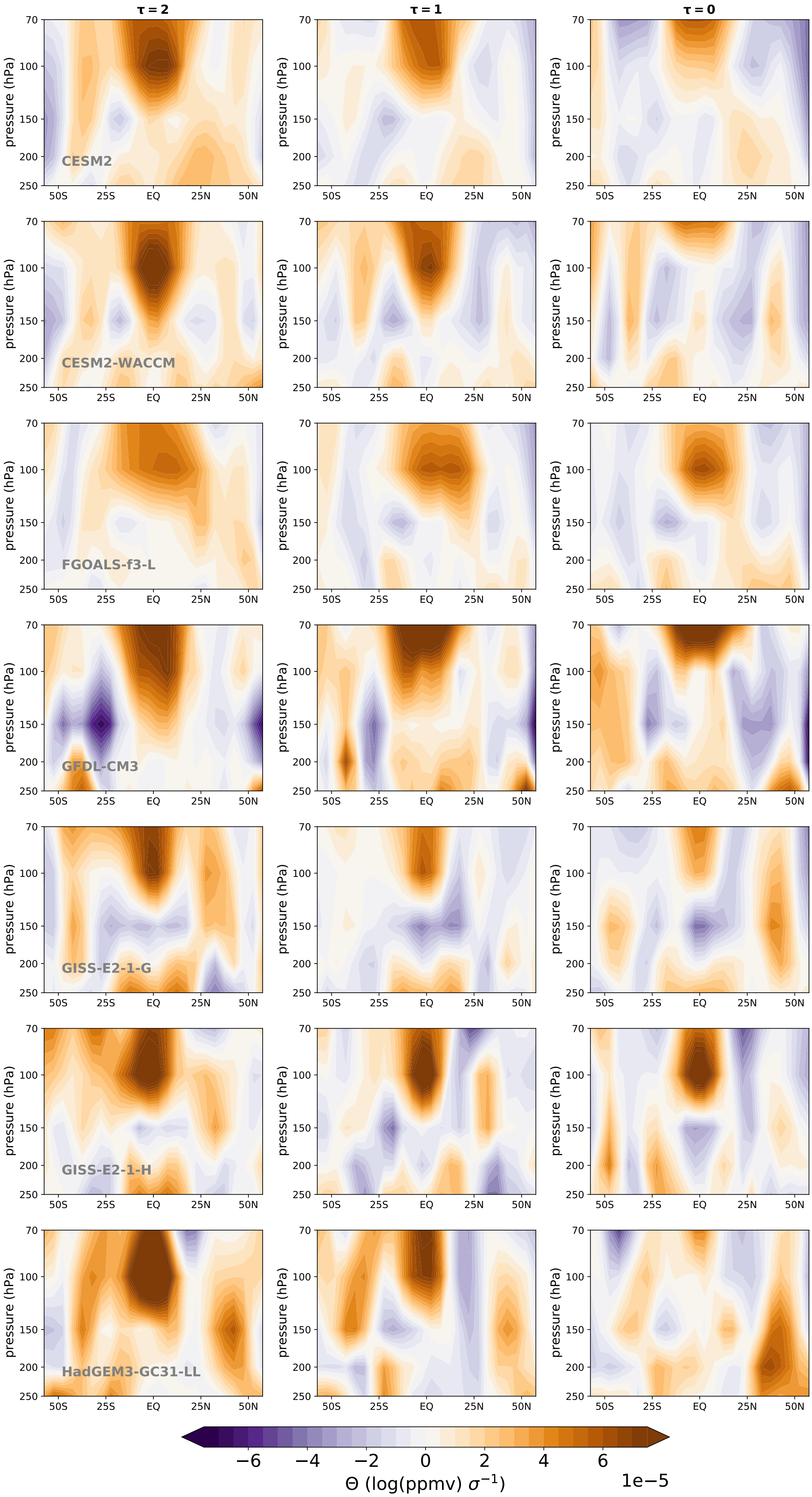

Supplementary Fig. 13 | Zonal mean cross sections of the ridge coefficients for individual CMIP models. As Fig. 4d in the main text, but for the second set of 8 of the 27 CMIP models (ordered alphabetically).

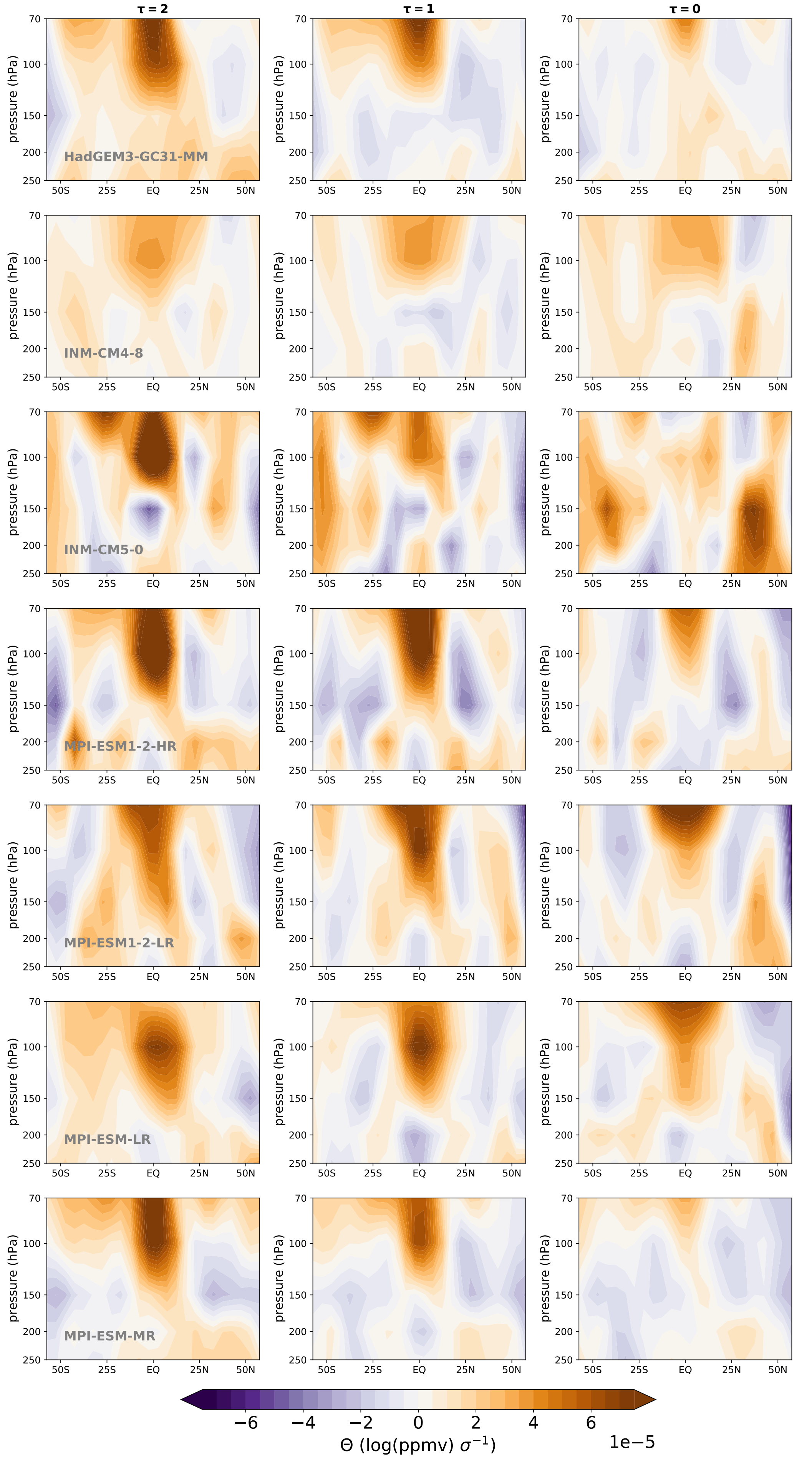

Supplementary Fig. 14 | Zonal mean cross sections of the ridge coefficients for individual CMIP models. As Fig. 4d in the main text, but for the third set of 8 of the 27 CMIP models (ordered alphabetically).

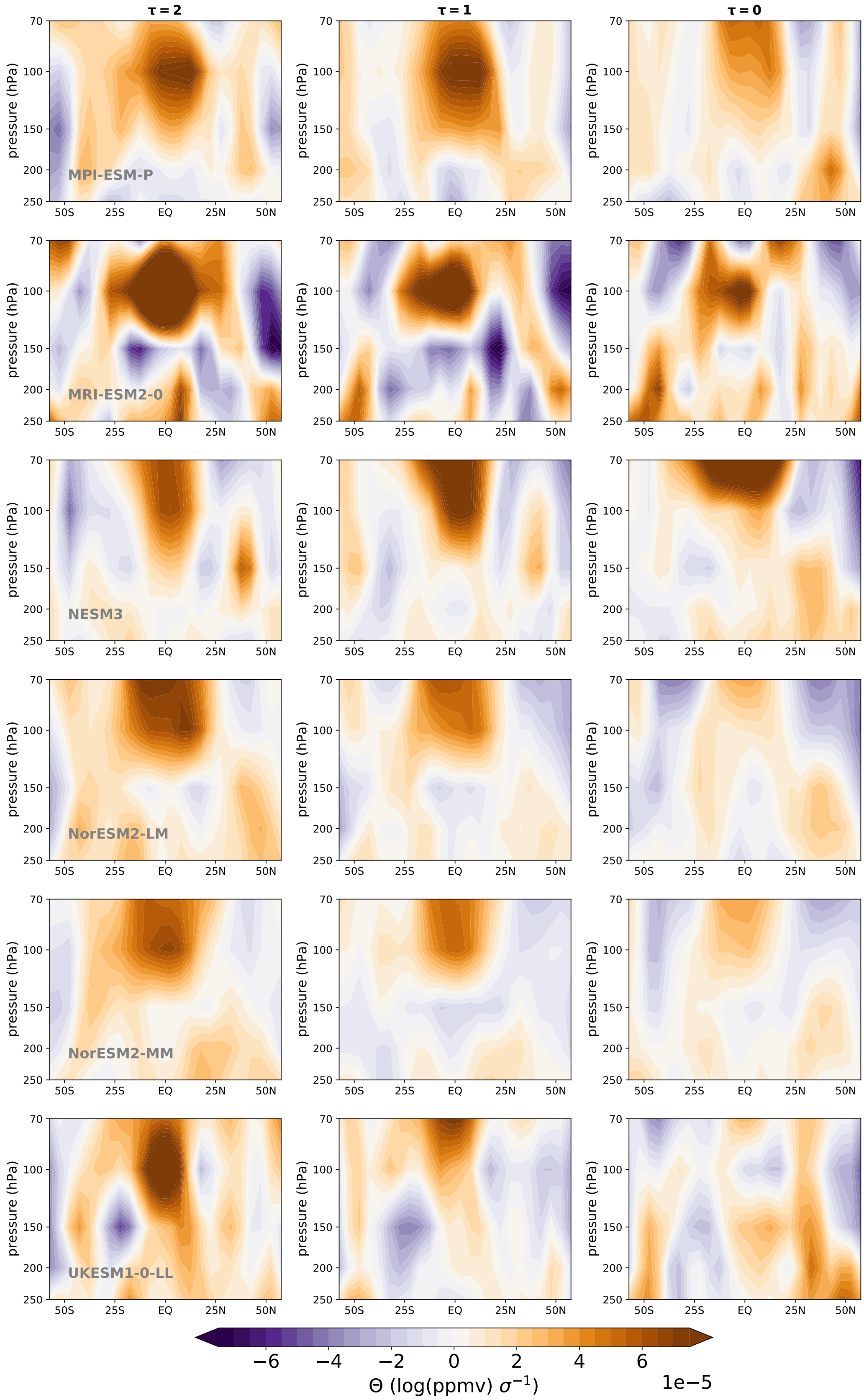

Supplementary Fig. 15 | Zonal mean cross sections of the ridge coefficients for individual CMIP models. As Fig. 4d in the main text, but for the final set of 7 of the 27 CMIP models (ordered alphabetically).

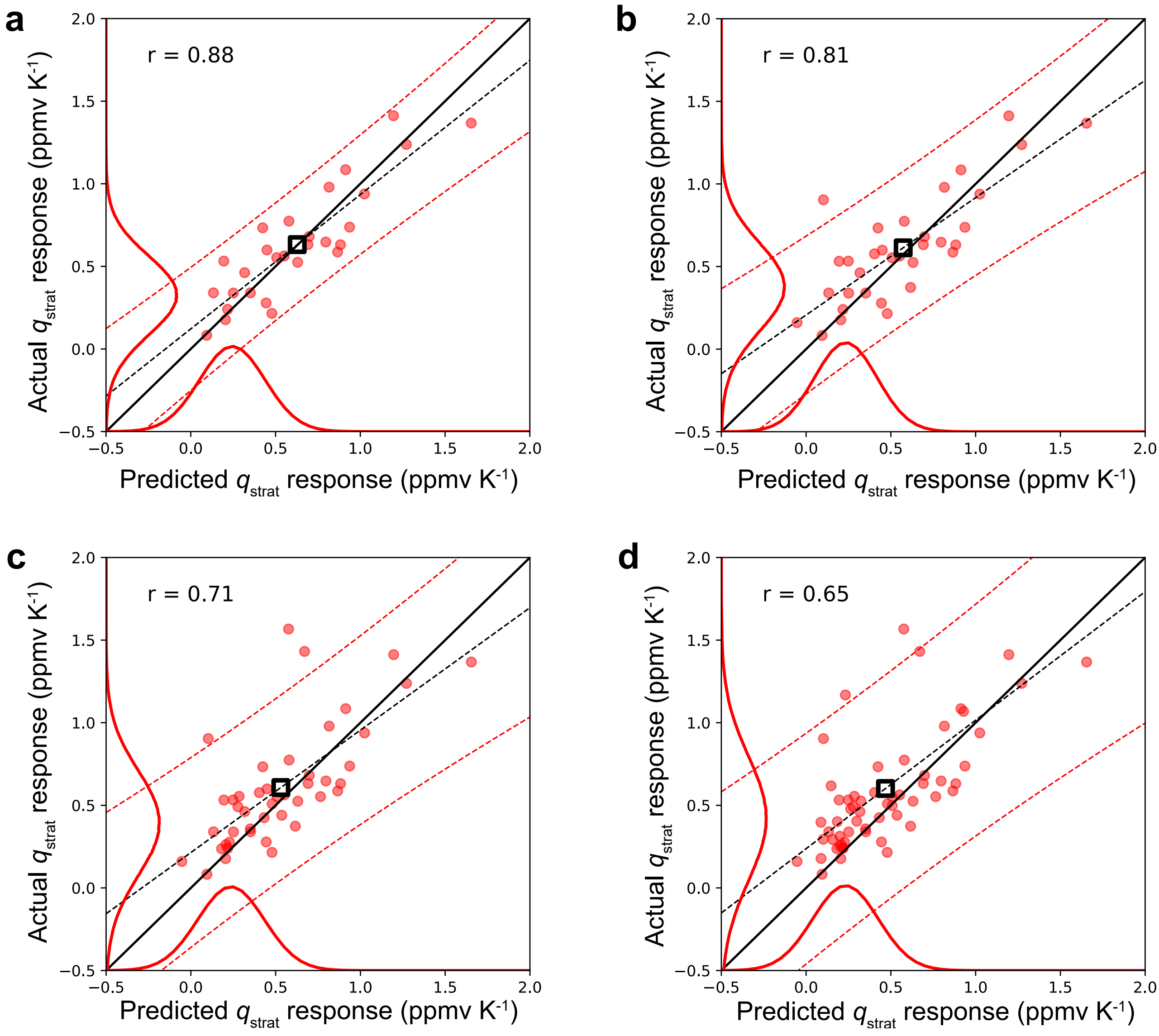

Supplementary Fig. 16 | Framework performance depending on the percentage of observational variance threshold set for CMIP model selection. As Fig. 2a in the main text, i.e. red circles show abrupt-4xCO_2 simulation results ('actual') regressed against predicted changes in q_{strat}, both normalized by T_g. The multi-model-mean is indicated as black square; the one-to-one line in solid black. Dashed lines show the least squares regression fit (black) and the 5 to 95% prediction intervals (red). The probability distributions (solid red curves) on the axes represent the observational estimates, with their amplitudes scaled arbitrarily. The distribution on top of the x-axis indicates the spread in normalized q_{strat} predictions based on combining functions learned from observations with the CMIP temperature responses. The final probability distribution, defining the observational constraint, is attached to the y-axis and additionally accounts for the uncertainty introduced by the statistical learning framework. **a** shows the results if only CMIP models are used for validation of the framework whose variance in q_{strat} matches 90% of the observed variance in SWOOSH, as is the case for 28 of the models. **b** shows the case where the threshold is lowered to 80% of the SWOOSH variance, which includes 33 models. **c** shows the case where the threshold is lowered substantially to 50% of the SWOOSH variance, which includes 45 models. The extrapolation performance decreases, which is reflective of large 4xCO_2 responses being hard to predict if training ridge regression on data that showed little variance in the predictand, a known issue when training ridge regression used for extrapolation (60; 61). Finally, **d** shows the case where all 61 CMIP5 and CMIP6 models are naively considered, equivalent to a selection threshold of 0% of observed variance. These results underline the need that, to obtain a realistic performance evaluation of the framework, it is necessary to screen the CMIP archive for models able to capture a sufficiently large fraction of the observed variance in q_{strat}.

References

[1] Bi, D. et al. The ACCESS Coupled Model: Description, Control Climate and Preliminary Validation. Australian Meteorological and Oceanographic Journal **63**, 41–64 (2013).

[2] Dix, M. et al. The ACCESS coupled model: documentation of core CMIP5 simulations and initial results. Australian Meteorological and Oceanographic Journal **63**, 83–99 (2013).

[3] Bi, D. et al. Configuration and spin-up of ACCESS-CM2, the new generation Australian Community Climate and Earth System Simulator Coupled Model. Journal of Southern Hemisphere Earth Systems Science **70**, 225–251 (2020).

[4] Ziehn, T. et al. The Australian Earth System Model: ACCESS-ESM1.5. Journal of Southern Hemisphere Earth Systems Science **70**, 193–214 (2020).

[5] Semmler, T. et al. Simulations for CMIP6 With the AWI Climate Model AWI-CM-1-1. Journal of Advances in Modeling Earth Systems **12**, e2019MS002009 (2020).

[6] Xin, X.-G. et al. How Well does BCC-CSM1.1 Reproduce the 20th Century Climate Change over China? Atmospheric and Oceanic Science Letters **6**, 21–26 (2013).

[7] Wu, T. et al. An Overview of BCC Climate System Model Development and Application for Climate Change Studies. Journal of Meteorological Research **28**, 34–56 (2014).

[8] Wu, T. et al. The Beijing Climate Center Climate System Model (BCC-CSM): The main progress from CMIP5 to CMIP6. Geoscientific Model Development **12**, 1573–1600 (2019).

[9] Wu, T. et al. Beijing Climate Center Earth System Model version 1 (BCC-ESM1): Model description and evaluation of aerosol simulations. Geoscientific Model Development **13**, 977–1005 (2020).

[10] Ji, D. et al. Description and basic evaluation of Beijing Normal University Earth System Model (BNU-ESM) version 1. Geoscientific Model Development **7**, 2039–2064 (2014).

[11] Rong, X.-Y. et al. Introduction of CAMS-CSM model and its participation in CMIP6. Climate Change Research **15**, 1673–1719 (2019).

[12] Von Salzen, K. et al. The Canadian fourth generation atmospheric global climate model (CanAM4). Part I: Representation of physical processes. Atmosphere - Ocean **51**, 104–125 (2013).

[13] Swart, N. C. et al. The Canadian Earth System Model version 5 (CanESM5.0.3). Geoscientific Model Development **12**, 4823–4873 (2019).

[14] Gent, P. R. et al. The Community Climate System Model Version 4. Journal of Climate **24**, 4973–4991 (2011).

[15] Danabasoglu, G. et al. The Community Earth System Model Version 2 (CESM2). Journal of Advances in Modeling Earth Systems **12**, e2019MS001916 (2020).

[16] Voldoire, A. et al. The CNRM-CM5.1 global climate model: Description and basic evaluation. Climate Dynamics **40**, 2091–2121 (2013).

[17] Voldoire, A. et al. Evaluation of CMIP6 DECK Experiments With CNRM-CM6-1. Journal of Advances in Modeling Earth Systems **11**, 2177–2213 (2019).

[18] Séférian, R. et al. Evaluation of CNRM Earth System Model, CNRM-ESM2-1: Role of Earth System Processes in Present-Day and Future Climate. Journal of Advances in Modeling Earth Systems **11**, 4182–4227 (2019).

[19] Jeffrey, S. et al. Australia's CMIP5 submission using the CSIRO-Mk3.6 model. Australian meteorological and Oceanographic Journal **63**, 1–13 (2013).

[20] Golaz, J. C. et al. The DOE E3SM Coupled Model Version 1: Overview and Evaluation at Standard Resolution. Journal of Advances in Modeling Earth Systems **11**, 2089–2129 (2019).

[21] Döscher, R. et al. The EC-Earth3 Earth system model for the Coupled Model Intercomparison Project 6. Geoscientific Model Development **15**, 2973–3020 (2022).

[22] Hazeleger, W. et al. EC-Earth V2.2: Description and validation of a new seamless earth system prediction model. Climate Dynamics **39**, 2611–2629 (2012).

[23] He, B. et al. CAS FGOALS-f3-L Model Datasets for CMIP6 Historical Atmospheric Model Intercomparison Project Simulation. Advances in Atmospheric Sciences **36**, 771–778 (2019).

[24] Li, L. et al. The Flexible Global Ocean-Atmosphere-Land System Model, Grid-Point Version 2: FGOALS-g2. Flexible Global Ocean-Atmosphere-Land System Model: Grid-point Version 2: FGOALS-g2 **30**, 543–560 (2013).

[25] Pu, Y. et al. CAS FGOALS-g3 Model Datasets for the CMIP6 Scenario Model Intercomparison Project (ScenarioMIP). Advances in Atmospheric Sciences **37**, 1081–1092 (2020).

[26] Griffies, S. M. et al. The GFDL CM3 coupled climate model: Characteristics of the ocean and sea ice simulations. Journal of Climate **24**, 3520–3544 (2011).

[27] Held, I. M. et al. Structure and Performance of GFDL's CM4.0 Climate Model. Journal of Advances in Modeling Earth Systems **11**, 3691–3727 (2019).

[28] Dunne, J. P. et al. GFDL's ESM2 Global Coupled Climate-Carbon Earth System Models. Part I: Physical Formulation and Baseline Simulation Characteristics. Journal of Climate **25**, 6646–6665 (2012).

[29] Dunne, J. P. et al. The GFDL Earth System Model Version 4.1 (GFDL-ESM 4.1): Overall Coupled Model Description and Simulation Characteristics. Journal of Advances in Modeling Earth Systems **12**, e2019MS002015 (2020).

[30] Kelley, M. et al. GISS-E2.1: Configurations and Climatology. Journal of Advances in Modeling Earth Systems **12**, e2019MS002025 (2020).

[31] Schmidt, G. A. et al. Configuration and assessment of the GISS ModelE2 contributions to the CMIP5 archive. Journal of Advances in Modeling Earth Systems **6**, 141–184 (2014).

[32] Jones, C. D. et al. The HadGEM2-ES implementation of CMIP5 centennial simulations. Geoscientific Model Development **4**, 543–570 (2011).

[33] Andrews, M. B. et al. Historical Simulations With HadGEM3-GC3.1 for CMIP6. Journal of Advances in Modeling Earth Systems **12**, e2019MS001995 (2020).

[34] Volodin, E. M., Dianskii, N. A. & Gusev, A. V. Simulating present-day climate with the INMCM4.0 coupled model of the atmospheric and oceanic general circulations. Izvestiya, Atmospheric and Oceanic Physics **46**, 414–431 (2010).

[35] Volodin, E. M., Evgeny V. Mortikov, Sergey V. Kostrykin, Vener Ya. Galin, Vasily N. Lykossov, Andrey S. Gritsun, Nikolay A. Diansky, Anatoly V. Gusev, Nikolay G. Iakovlev, A. A. S. & Emelina, S. V. Simulation of the modern climate using the INM-CM48 climate model. Russian Journal of Numerical Analysis and Mathematical Modelling **December** (2018).

[36] Volodin, E. & Gritsun, A. Simulation of observed climate changes in 1850-2014 with climate model INM-CM5. Earth System Dynamics **9**, 1235–1242 (2018).

[37] Dufresne, J. L. et al. Climate change projections using the IPSL-CM5 Earth System Model: From CMIP3 to CMIP5. Climate Dynamics **40**, 2123–2165 (2013).

[38] Boucher, O. et al. Presentation and Evaluation of the IPSL-CM6A-LR Climate Model. Journal of Advances in Modeling Earth Systems **12**, e2019MS002010 (2020).

[39] Watanabe, M. et al. Improved Climate Simulation by MIROC5: Mean States, Variability, and Climate Sensitivity. Journal of Climate **23**, 6312–6335 (2010).

[40] Tatebe, H. et al. Description and basic evaluation of simulated mean state, internal variability, and climate sensitivity in MIROC6. Geoscientific Model Development **12**, 2727–2765 (2019).

[41] Hajima, T. et al. Development of the MIROC-ES2L Earth system model and the evaluation of biogeochemical processes and feedbacks. Geoscientific Model Development **13**, 2197–2244 (2020).

[42] Watanabe, S. et al. MIROC-ESM: model description and basic results of CMIP5-20c3m experiments. Geoscientific Model Development Discussions **4**, 1063–1128 (2011).

[43] Gutjahr, O. et al. Max Planck Institute Earth System Model (MPI-ESM1.2) for the High-Resolution Model Intercomparison Project (HighResMIP). Geoscientific Model Development **12**, 3241–3281 (2019).

[44] Müller, W. A. et al. A Higher-resolution Version of the Max Planck Institute Earth System Model (MPI-ESM1.2-HR). Journal of Advances in Modeling Earth Systems **10**, 1383–1413 (2018).

[45] Mauritsen, T. et al. Developments in the MPI-M Earth System Model version 1.2 (MPI-ESM1.2) and Its Response to Increasing CO2. Journal of Advances in Modeling Earth Systems **11**, 998–1038 (2019).

[46] Giorgetta, M. A. et al. Climate and carbon cycle changes from 1850 to 2100 in MPI-ESM simulations for the Coupled Model Intercomparison Project phase 5. Journal of Advances in Modeling Earth Systems **5**, 572–597 (2013).

[47] Yukimoto, S. et al. A New Global Climate Model of the Meteorological Research Institute: MRI-CGCM3. Journal of the Meteorological Society of Japan **90A**, 23–64 (2012).

[48] Yukimoto, S. et al. The Meteorological Research Institute Earth System Model Version 2.0, MRI-ESM2.0: Description and Basic Evaluation of the Physical Component. Journal of the Meteorological Society of Japan **97**, 931–965 (2019).

[49] Cao, J. et al. The NUIST Earth System Model (NESM) version 3: Description and preliminary evaluation. Geoscientific Model Development **11**, 2975–2993 (2018).

[50] Iversen, T. et al. The Norwegian Earth System Model, NorESM1-M – Part 2: Climate response and scenario projections. Geoscientific Model Development **6**, 389–415 (2013).

[51] Seland, Ø. et al. Overview of the Norwegian Earth System Model (NorESM2) and key climate response of CMIP6 DECK, historical, and scenario simulations. Geoscientific Model Development **13**, 6165–6200 (2020).

[52] Park, S., Shin, J., Kim, S., Oh, E. & Kim, Y. Global climate simulated by the Seoul National University Atmosphere Model version 0 with a unified convection scheme (SAM0-UNICON). Journal of Climate **32**, 2917–2949 (2019).

[53] Sellar, A. A. et al. Implementation of U.K. Earth System Models for CMIP6. Journal of Advances in Modeling Earth Systems **12**, e2019MS001946 (2020).

[54] Nowack, P. J., Braesicke, P., Abraham, N. L. & Pyle, J. A. On the role of ozone feedback in the ENSO amplitude response under global warming. Geophysical Research Letters **44**, 3858–3866 (2017).

[55] Dessler, A. E., Schoeberl, M. R., Wang, T., Davis, S. M. & Rosenlof, K. H. Stratospheric water vapor feedback. Proceedings of the National Academy of Sciences of the United States of America **110**, 18087–91 (2013).

[56] Dessler, A. E. et al. Variations of stratospheric water vapor over the past three decades. Journal of Geophysical Research : Atmospheres **119**, 12588–12598 (2014).

[57] Smalley, K. M. et al. Contribution of different processes to changes in tropical lower-stratospheric water vapor in chemistry-climate models. Atmospheric Chemistry and Physics **17**, 8031–8044 (2017).

[58] Taylor, K. E., Stouffer, R. J. & Meehl, G. A. An overview of CMIP5 and the experiment design. Bulletin of the American Meteorological Society **93**, 485–498 (2012).

[59] Eyring, V. et al. Overview of the Coupled Model Intercomparison Project Phase 6 (CMIP6) experimental design and organization. Geoscientific Model Development **9**, 1937–1958 (2016).

[60] Nowack, P. et al. Using machine learning to build temperature-based ozone parameterizations for climate sensitivity simulations. Environmental Research Letters **13**, 104016 (2018).

[61] Nowack, P., Konstantinovskiy, L., Gardiner, H. & Cant, J. Machine learning calibration of low-cost NO2 and PM10 sensors: non-linear algorithms and their impact on site transferability. Atmospheric Measurement Techniques **14**, 5637–5655 (2021).

[62] Ceppi, P. & Nowack, P. Observational evidence that cloud feedback amplifies global warming. Proc. Natl. Acad. Sci. USA **118**, e2026290118 (2021).
